# A DNA vaccine candidate delivered by an electroacupuncture machine provides protective immunity against SARS-CoV-2 infection

**DOI:** 10.1038/s41541-022-00482-0

**Published:** 2022-06-03

**Authors:** Tsai-Teng Tzeng, Kit Man Chai, Kuan-Yin Shen, Chia-Yi Yu, Shiu-Ju Yang, Wan-Chun Huang, Hung-Chun Liao, Fang-Feng Chiu, Horng-Yunn Dou, Ching-Len Liao, Hsin-Wei Chen, Shih-Jen Liu

**Affiliations:** 1 National Institute of Infectious Diseases and Vaccinology, National Health Research Institutes, Miaoli, Taiwan; 2 Department of Life Sciences, National Tsing Hua University, Hsinchu, Taiwan; 3 Graduate Institute of Biomedical Sciences, China Medical University, Taichung, Taiwan; 4 Graduate Institute of Medicine, College of Medicine, Kaohsiung Medical University, Kaohsiung, Taiwan

**Keywords:** Viral infection, DNA vaccines

## Abstract

A major challenge in the use of DNA vaccines is efficient DNA delivery in vivo. Establishing a safe and efficient electric transfer method is the key to developing rapid DNA vaccines against emerging infectious diseases. To overcome the complexity of designing new electric transfer machines for DNA delivery, a clinically approved electric transfer machine could be considered as an alternative. Here, we report an electroacupuncture machine-based method for DNA vaccine delivery after intramuscular injection of the COVID-19 DNA vaccine. The S gene of SARS-CoV-2 in the pVAX1 plasmid (pSARS2-S) was used as an antigen in this study. We optimized the clinically used electroacupuncture machine settings for efficient induction of the neutralizing antibody titer after intramuscular injection of pSARS2-S in mice. We found that pSARS2-S immunization at 40 Vpp for 3–5 s could induce high neutralizing antibody titers and Th1-biased immune responses. IFN-γ/TNF-α-secreting CD4^+^ and CD8^+^ T cells were also observed in the DNA vaccination group but not in the recombinant protein vaccination group. T-cell epitope mapping shows that the major reactive epitopes were located in the N-terminal domain (a.a. 261–285) and receptor-binding domain (a.a. 352–363). Importantly, pSARS2-S immunization in hamsters could induce protective immunity against SARS-CoV-2 challenge in vivo. In the preclinical toxicology study, blood biochemistry, hematology, and DNA persistence analysis reveal that the DNA delivery method is safe. Furthermore, the raised antisera could also cross-neutralize different variants of concern. These findings suggest that DNA vaccination using an electroacupuncture machine is feasible for use in humans in the future.

## Introduction

More than 372 million people have been infected with SARS-CoV-2, and more than 5.65 million people have died globally as of January 2022 since the outbreak of COVID-19 began^1^. Although some vaccines have become available for use in humans, additional different types of vaccines are still under development. Nucleic acid-based vaccines, especially mRNA vaccines, have been recognized as next-generation vaccines. However, the ultralow-temperature storage of mRNA limits its delivery at a global scale. In contrast, DNA is very stable under refrigeration or even at room temperature. DNA vaccine development could be another hot spot for research. Recently, in August 2021, a needle-free COVID-19 DNA vaccine was approved for emergency use authorization (EUA) in India^2^. DNA vaccines have several advantages, including rapid production, low sensitivity to temperature, and low cost. Hence, the use of DNA vaccines is a potential approach to combat emergent infectious disease outbreaks. Currently, more than 17 clinical studies of DNA-based vaccines for COVID-19 are registered in ClinicalTrials.gov. Most of them use electric transfer to increase the efficiency of DNA vaccines, suggesting that electric transfer to deliver DNA is still the main trend. Electroporation (EP) combined with DNA vaccination greatly increases the efficacy of DNA vaccines^3–5^. Although there are different EP devices developed for human use, including Cellectra^®^ (Inovio Inc.), Trigrid^®^ (Ichor Medical Systems), and Cliniporator^®^ (IGEA medical)^6–8^, it may raise safety concerns from regulatory authorities about the use of newly developed EP device, and the process can be time consuming. To overcome this limitation, a clinically available electroacupuncture machine was used in our DNA vaccination study for COVID-19. Experience gained from electroacupuncture may contribute to its new intended use as a DNA vaccine delivery system with fewer safety concerns. Instead of developing complex components for the delivery, we developed a simple cartridge to assemble commercial injection syringe with 2-needle array, and connect with the electroacupuncture for clinical uses. Therefore, this delivery approach could accelerate the development of DNA vaccine platform during emergency outbreak.

Acupuncture has been used for a long time in China and worldwide. In the USA, ~3.5 million adults receive acupuncture therapy each year^9^. Electroacupuncture machines deliver a lower voltage (5–40 peak-to-peak voltage (Vpp)) than other DNA vaccine devices (40–200 V/pulse for Cellectra^®^; 250 V/cm for Trigrid^®^ (the electrode array was in a diamond-shaped configuration with 6 mm electrode interval))^7,10^. Electroacupuncture has been shown to regulate immune responses by activating the JAK2/STAT3 signaling pathway in macrophages^11^ or activating the vagus nerve to enhance antitumor immunity^12^. These results indicated that electric transfer from the electroacupuncture machine not only increases DNA uptake but also activates immune cells to secrete cytokines to enhance immune responses. Furthermore, electric pulses may induce inflammatory responses and may facilitate DNA vaccine efficacy in vivo.

Our previous studies showed that a DNA vaccine with the tumor-associated antigen L6 followed by EP could induce cellular immunity against cancer growth^13^. We further applied this approach for COVID-19, and the DNA-encoded full-length spike (S) protein of SARS-CoV-2 (pSARS2-S) was shown to induce protective immune responses against viral challenge in hamsters^14^. EP combined with DNA vaccination greatly increases the efficacy of DNA vaccines^3–5^. In this study, we examined DNA vaccination with an electroacupuncture machine that could induce neutralizing antibody and Th1-biased immune responses in both mice and rats. The T cell epitopes were analyzed using overlapping synthetic peptides. The raised antisera could neutralize different variants of SARS-CoV-2. Immunized hamsters showed induction of protective immunity against SARS-CoV-2 challenge.

## Results

### Optimization of EP conditions using an electroacupuncture machine

We have previously applied an EP device for use in animals to deliver the SARS-CoV-2 DNA vaccine, which has a protective effect against SARS-CoV-2 infection in hamsters^14^. A suitable delivery system to enhance DNA vaccine efficacy is required to bridge the gap between the development phase and clinical study. To this end, we applied a clinically approved electroacupuncture machine to accelerate the development of our SARS-COV-2 DNA vaccine for use in preclinical and clinical studies. To optimize electrical stimulation conditions to deliver the DNA vaccine, BALB/c mice were intramuscularly immunized twice at a 3-week interval with vector and pSARS2-S DNA, followed by EP using the electroacupuncture machine under different Vpp, total stimulation times, or amounts of DNA.

**Fig. 1:**
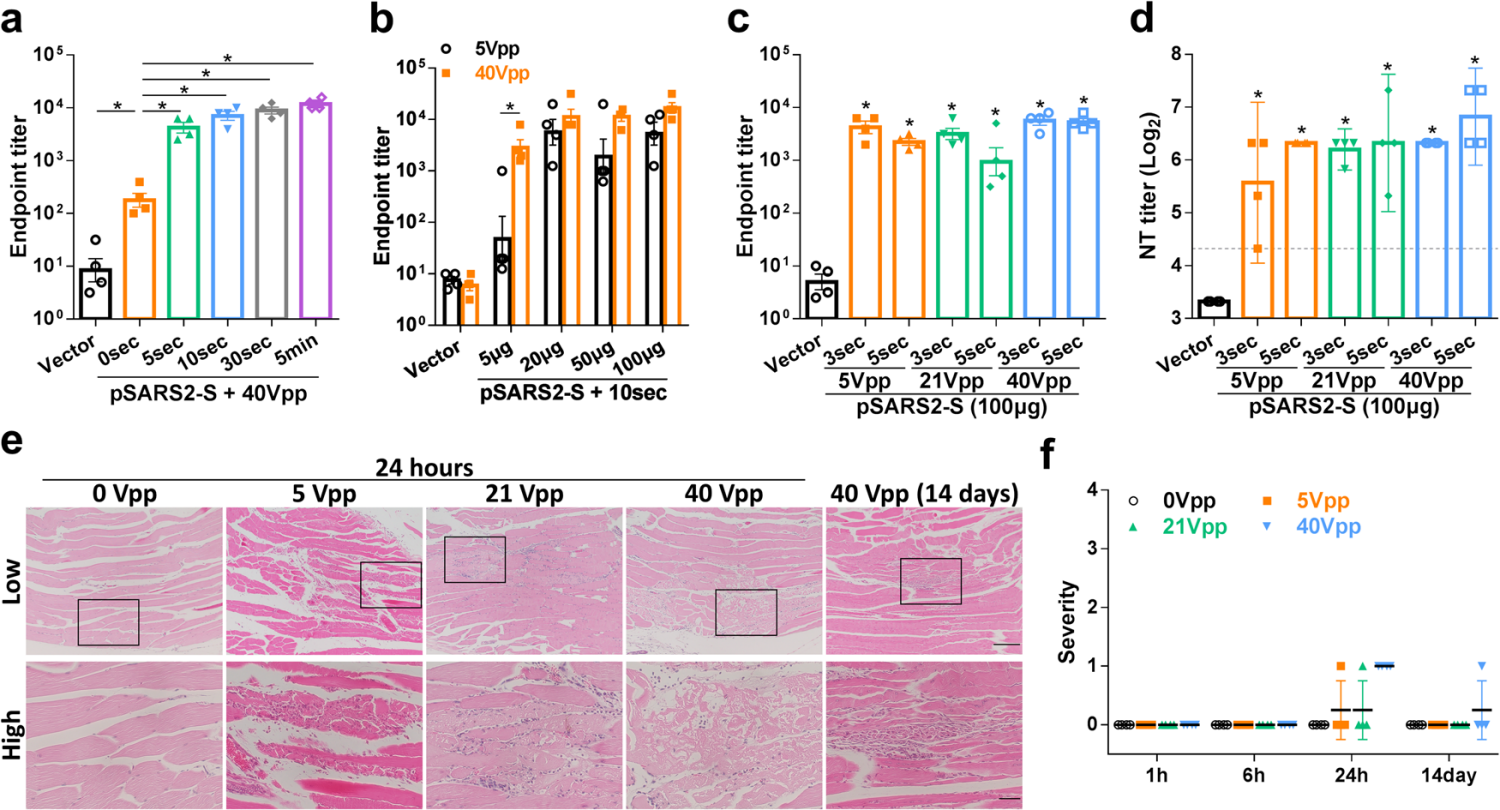
Optimization of electrostimulation conditions using electroacupuncture machine. BALB/c mice (*n* = 4 per group) were intramuscularly immunized twice at a 3-week interval with vector or pSARS2-S DNA, followed by electrostimulation by an electroacupuncture machine under different peak-to-peak voltages (Vpp), total stimulation times, or amounts of DNA. Serum samples were collected at week 6 after the first immunization. **a**–**c** IgG antibodies against spike protein were assessed by ELISA. **d** Vaccine‐induced neutralizing activity against wild-type SARS-CoV-2 was evaluated by neutralization assay. The dashed line indicates the initial fold dilution of serum samples. **e**, **f** BALB/c mice were electrostimulated with 0, 5, 21, and 40 Vpp for 5 s. At the indicated timepoints after electrostimulation, muscle tissues at the injection site (gastrocnemius muscle) were collected and examined by hematoxylin and eosin staining. **e** Representative images of inflammation in the muscle tissue. Enlarged views of the area in the black box are shown at the bottom. Scale bar for low-magnification: 200 μm, for high-magnification: 50 μm. **f** The inflammatory severity score was evaluated based on the percentage of the inflammatory area. Antibody titers and severity scores are presented as the mean ± SEM, and neutralization titers are expressed as the geometric mean with a 95% confidence interval. *p < 0.05 by the Mann–Whitney test.

To evaluate the effects of different stimulation times on vaccine efficacy, BALB/c mice were injected with 100 μg of DNA vaccine and immediately pulsed with 40 Vpp electrical stimulation for 0, 5, 10, 30 s or 5 min. After two doses of DNA immunization, all groups with pSARS2-S immunization induced higher antibody titers against spike protein than the vector control group (Fig. 1a). The log titers of the stimulation groups ranged from 3.4 to 4.2, which was markedly higher than the 2.25 titer elicited by pSARS2-S without electrostimulation (0-s group). Notably, electrical stimulation for as little as 5 s may be sufficient to enhance DNA vaccine efficacy to the level induced by longer (30 s and 5 min) stimulation.

Moreover, we evaluated the minimum effective DNA dose required for vaccination under various electrical stimulation voltages. BALB/c mice were injected with 5 to 100 μg of DNA vaccine and immediately pulsed with 5 or 40 Vpp electrical stimulation for 10 s. Under 5 Vpp stimulation, 20–100 μg of DNA vaccine clearly induced antibody titers, but this was not observed in the 5 μg DNA group (Fig. 1b). In contrast, sera from mice pulsed by 40 Vpp stimulation showed clear antibody titers in the 5 μg DNA group. Likewise, 40 Vpp stimulation induced similar antibody levels in the 20–100 μg DNA groups, which was similar to the levels elicited by 5 Vpp stimulation. These results indicated that stimulation voltages of 5–40 Vpp effectively improved the immunogenicity of the DNA vaccine, but more DNA (20–100 μg) was required to elicit sufficient vaccine efficacy under stimulation with as low as 5 Vpp.

To minimize tissue damage without compromising DNA vaccine immunogenicity, we further shortened the electrostimulation time to 3 s and decreased the voltage to 5 and 21 Vpp. The results showed that pSARS2-S immunization with 3- and 5-s stimulations elicited similar antibody titers against spike protein, regardless of voltage level (Fig. 1c). Notably, IgG antibody titers elicited by pSARS2-S immunization with electrostimulation persisted for at least 16 weeks after the first immunization (Supplementary Fig. 1a), suggesting that electroacupuncture machine-based DNA vaccine delivery could induce a long-lasting humoral response against SARS-CoV-2. Neutralizing antibody analysis against SARS-CoV-2 further showed that either 21 or 40 Vpp stimulation for 3 or 5 s induced similar levels of neutralizing antibodies (Fig. 1d). In the 5 Vpp stimulation groups, sera from the 5-s group had neutralization activity similar to those elicited by 21 and 40 Vpp stimulation. The geometric neutralization titer of the 3-s group with 5 Vpp stimulation was 47.6, but one mouse serum from this group did not achieve a 4-fold rise relative to the vector control group. Taken together, the results showed that 21–40 Vpp electrostimulation for 3–5 s was enough to induce antibody response against SARS-CoV-2, and 5 Vpp stimulation for up to 5 s was required to induce sufficient vaccine efficacy.

Due to safety concerns, we performed an electrostimulation safety test to evaluate local tissue response at 1, 6, 24 h, and 14 days after electrostimulation with various voltage conditions (0, 5, 21 and 40 Vpp). Histological analysis of muscle showed that there was no tissue alteration in any groups at 1 and 6 h (Fig. 1f). Minimal to mild necrosis and minimal inflammation of muscle were found in one animal in the 5 and 21 Vpp groups and all animals in the 40 Vpp group at 24 h, suggesting that incidence and severity increased with voltage strength (Fig. 1e, f). After a 14-day recovery period, muscle regeneration was observed in one mouse in the 40 Vpp group (Fig. 1e), and no fibrosis was observed in any groups. These results indicated that only minimal to mild damage was caused by electrostimulation delivered by the electroacupuncture machine, and this damage was considered to be reversible after 14 days.

### Induction of Th1- or Th2-biased responses

**Fig. 2:**
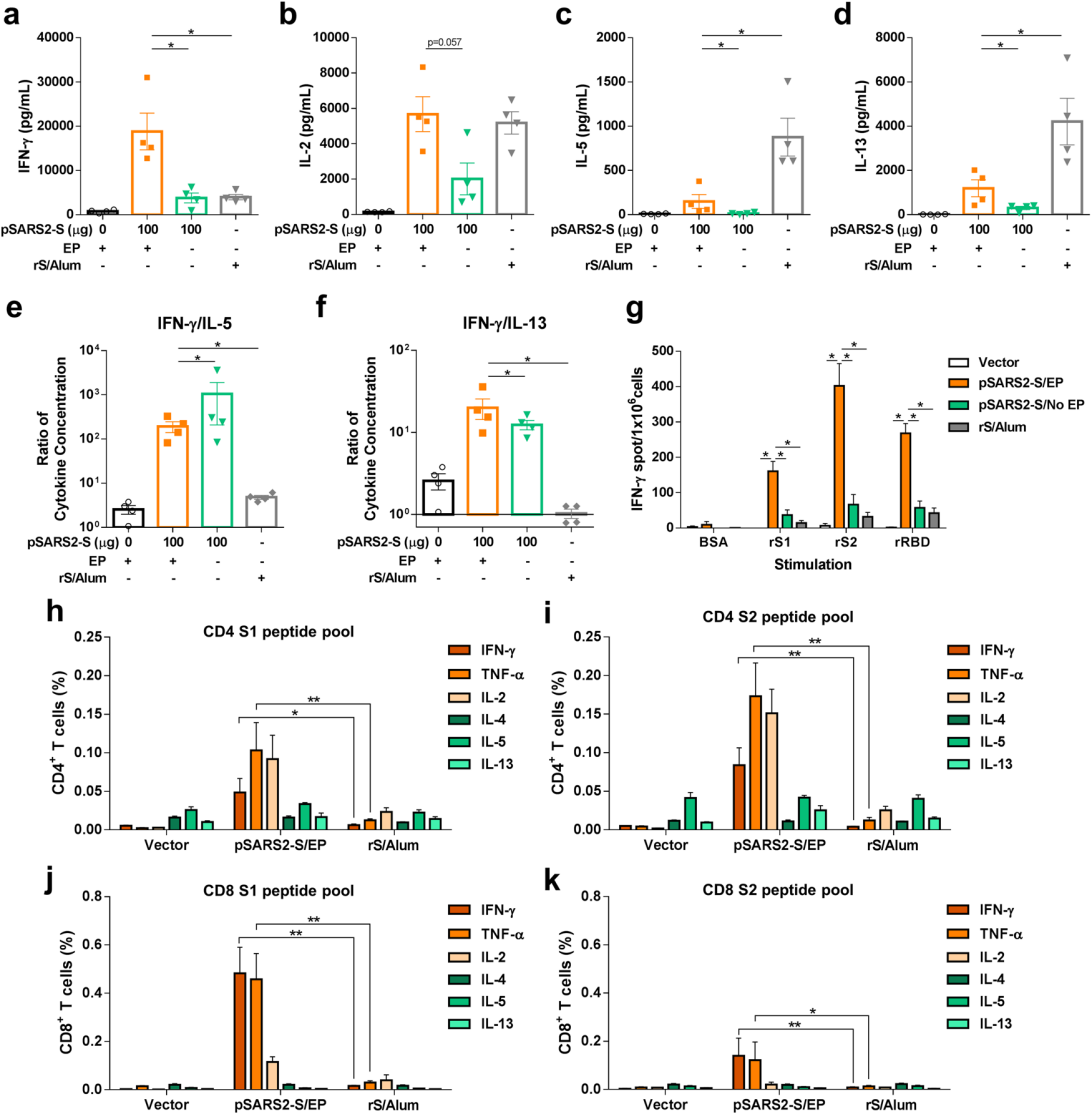
T cell responses in mice after immunization with pSARS2-S DNA vaccine. BALB/c mice (*n* = 4 per group) were intramuscularly immunized twice at a 3-week interval with 100 μg of vector, 100 μg of pSARS2-S with 40 Vpp electrostimulation for 5 s (pSARS2-S/EP), pSARS2-S without electrostimulation (pSARS2-S/No EP), or 10 μg of recombinant spike protein adjuvanted with 150 μg aluminum hydroxide (rS/Alum). Splenocytes were collected at week 5 after the first immunization, and the levels of secreted IFN-γ, IL-2, IL-5 and IL-13 (**a**–**d**) were evaluated after restimulation with recombinant spike protein. **e, f** The ratio of IFN-γ to IL-5 and the ratio of IFN-γ to IL-13 were calculated. **g** Specific IFN-γ-secreting T cells were also analyzed by IFN-γ ELISpot in splenocytes following stimulation with the indicated recombinant proteins for 48 h. **h**–**k** Cytokine expression of spike-specific CD4^+^ and CD8^+^ memory T cells. Splenocytes were collected as mentioned above and stimulated with two pools of overlapping peptides spanning the entire spike protein for 6 h. Analyzed cells were gated on CD44^hi^/cytokine^+^ for the memory T population. Percentage of memory CD4^+^ T cells producing IFN-γ, TNF-α, IL-2, IL-4, IL-5 and IL-13 in response to S1 (**h**) and S2 (**i**) peptide pools. Percentage of CD8^+^ memory T cells producing IFN-γ, TNF-α, IL-2, IL-4, IL-5 and IL-13 in response to the S1 (**j**) and S2 (**k**) peptide pools. The values are presented as the mean ± SEM. *p < 0.05, **p < 0.01 by the Mann–Whitney test.

We assessed T cell responses after the immunization of DNA vaccine with or without electrical stimulation (pSARS2-S/EP or pSARS2-S/No EP) or recombinant spike protein adjuvanted with alum (rS/Alum). Splenocytes from the immunized mice were stimulated with spike protein. The secretion of Th1-type cytokines (IFN-γ) and Th2-type cytokines (IL-5 and IL-13) was detected by ELISA. Vaccination with rS/Alum induced higher levels of the Th2-type cytokines IL-5 (876.0 ± 214.0 pg/mL) and IL-13 (4209.1 ± 1052.4 pg/mL), which was used as a control for the Th2-biased immune response (Fig. 2c, d). In contrast, the pSARS2-S/EP group elicited the highest amounts of IFN-γ (18810.0 ± 4140.0 pg/mL) and IL-2 (5673.6 ± 988.3 pg/mL) among all the groups (Fig. 2a, b). Furthermore, analysis of the Th1/Th2 ratio showed that the splenocytes from the pSARS2-S/EP and pSARS2-S/No EP groups secreted higher levels of the Th1-type cytokine IFN-γ than of the Th2-type cytokines IL-5 and IL-13 when compared with the control and rS/Alum groups (Fig. 2e, f). Consistent with this, the pSARS2-S/EP group induced more IFN-γ-secreting cells upon ex vivo stimulation with recombinant spike protein fragments (S1, S2 and receptor-binding domain (RBD)) than the rS/Alum group (Fig. 2g), as measured by ELISpot assay. Therefore, these results indicated that the pSARS2-S vaccine induced a Th1-biased immune response regardless of whether it was combined with electrical stimulation.

Apart from that, we also evaluated the cytokine profiles of CD4^+^ and CD8^+^ memory T cells elicited by DNA vaccination. Using intracellular cytokine staining combined with surface marker staining, we found that immunization of mice with pSARS2-S/EP elicited higher levels of Th1 cytokine IFN-γ- and TNF-α-secreting CD4^+^ and CD8^+^ T cells (Fig. 2h–k) in response to both the S1 and S2 peptide pools than immunization of mice with alum-adjuvanted spike protein. Furthermore, immunization with pSARS2-S/EP induced a significantly stronger CD8^+^ T cell response to the S1 peptide pool (Fig. 2j) than to the S2 peptide pool (Fig. 2k). Similar to a previous study on a nucleic acid-based SARS-CoV-2 vaccine (mRNA-1273)^15^, DNA vaccination with electrical stimulation triggered both CD4^+^ and CD8^+^ T cell responses.

### Identification of T cell epitopes after DNA vaccination

**Fig. 3:**
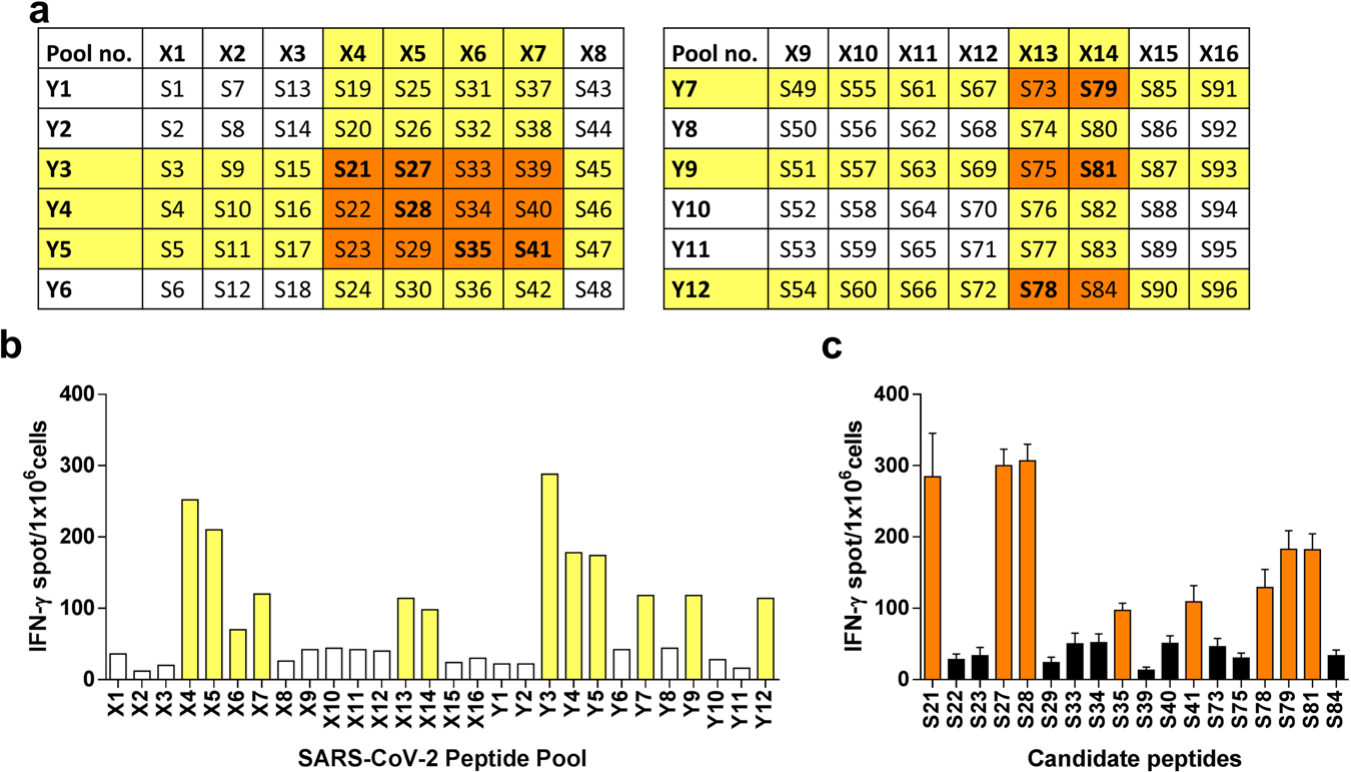
T cell epitope mapping after DNA vaccination using electroacupuncture machine. **a** Two peptide matrices contained sequential peptide pools X1–X16 and nonsequential peptide pools Y1–Y12. The amino acid sequence of each peptide is shown in Supplementary Data 1. **b** To identify the T cell epitopes of the spike protein, splenocytes from DNA vaccine-immunized mice (100 μg pSARS2-S/40 Vpp/120 Hz/5 s) were restimulated with each peptide pool for 48 h. IFN-γ-producing cells were determined by ELISpot. Based on the number of IFN-γ-producing cells, responsive stimulatory pools are marked yellow in the peptide matrices, and candidate peptides at the intersections of the stimulatory rows and columns are indicated in orange. **c** To identify the immunodominant T cell epitopes of the spike protein, splenocytes from immunized mice were restimulated with each candidate peptide individually. Antigenic peptides are presented in bold in the peptide matrices. The data are presented as the mean ± SEM.

To further identify T cell epitopes after two-dose pSARS2-S DNA vaccination, we synthesized 23–25 amino acid peptides with a 10-amino-acid overlap covering the entire spike protein sequence (Supplementary Data 1). Peptide pools were designed into matrices as shown in Fig. 3a. T cell epitope mapping was analyzed by IFN-γ-ELISpot in splenocytes pulsed with the peptide pools for 48 h, and several stimulatory peptide pools were detected (Fig. 3b). Next, candidate peptides at the intersections of the stimulatory peptide pools were further assessed to identify potential immunodominant T cell epitopes. As shown in Fig. 3c, peptides S21, S27, S28, S35, S41, S78, S79, and S81 could stimulate primed T cells to produce IFN-γ. These peptide sequences and amino acid positions in the spike protein are shown in Table 1. These identified immunodominant peptides were found in the N-terminal domain (S21), receptor-binding domain (S27 and S28) and S2 region (S78, S79 and S81). These results indicated that the T cell epitope elicited by the pSARS2-S DNA vaccine against SARS-CoV-2 was detected.

**Table 1:** Antigenic peptides of the spike protein.

Name	Position	Peptide sequence
S21	S_261-285_	GAAAYYVGYLQPRTFLLKYNENGTI
S27	S_339-363_	GEVFNATRFASVYAWNRKRISNCVA
S28	S_352-376_	AWNRKRISNCVADYSVLYNSASFST
S35	S_443-467_	SKVGGNYNYLYRLFRKSNLKPFERD
S41	S_520-545_	PATVCGPKKSTNLVKNKCVNFNFNG
S78	S_1003-1026_	SLQTYVTQQLIRAAEIRASANLAA
S79	S_1015-1039_	AAEIRASANLAATKMSECVLGQSKR
S81	S_1041-1065_	DFCGKGYHLMSFPQSAPHGVVFLHV

Underline indicated overlapping region in adjacent peptides (S27/S28 or S78/S79) containing potential T cell epitopes.

### DNA vaccination-induced protective effect against SARS-CoV-2 challenge

**Fig. 4:**
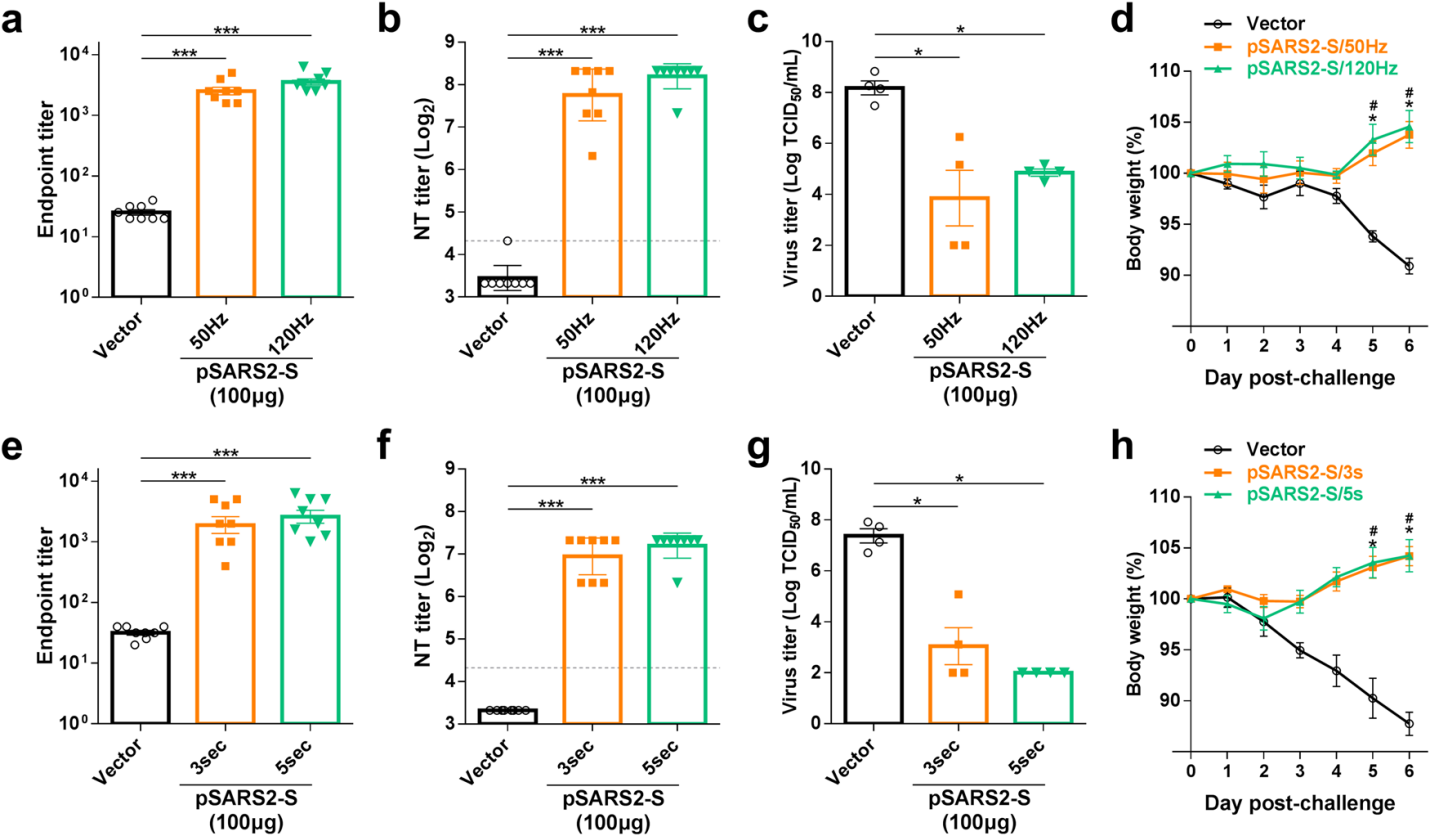
The effect of stimulation frequency and time on SARS-CoV-2-infected hamsters. **a**–**d** Syrian hamsters (*n* = 8 per group) were intramuscularly immunized three times at a 2-week interval with 100 μg of vector control or pSARS2-S, followed by electrostimulation at 40 Vpp with a frequency of 50 or 120 Hz for 10 s. **e**–**h** Hamsters (*n* = 8 per group) received 40 Vpp/120 Hz electrostimulation for 3 or 5 s following DNA injection, and three-dose vaccination regimen was described as above. Serum samples obtained at week 6 after the first immunization were used in this experiment. **a**, **e** Endpoint titers of serum IgG specific to spike protein were evaluated by ELISA. **b**, **f** Serum neutralizing activity against wild-type SARS-CoV-2 was evaluated by neutralization assay. The dashed line indicates the initial fold dilution of serum samples. At 3 weeks after the final immunization, Syrian hamsters were intranasally challenged with 10^5^ TCID_50_ SARS-CoV-2. **c**, **g** Viral titers in the lungs of SARS-CoV-2-infected hamsters at 3 days postchallenge were determined by TCID_50_ assay. **d**, **h** Body weight change (%) of the hamsters was recorded every day after SARS-CoV-2 challenge. Neutralization titers are expressed as the geometric mean with a 95% confidence interval, and other data are presented as the mean ± SEM. *p < 0.05, ***p < 0.001 by the Mann–Whitney test.

SARS-CoV-2 causes body weight loss and lung inflammation in Syrian hamsters^16,17^, which is a SARS-CoV-2 challenge model for the evaluation of COVID-19 vaccines^14,18,19^. Hence, we applied this model to evaluate the effect of stimulation frequency (50 or 120 Hz) on pSARS2-S vaccine immunogenicity and protection against SARS-CoV-2 infection. The groups immunized with pSARS2-S at 50 and 120 Hz stimulation frequencies induced higher spike-specific antibody titers and neutralizing antibody titers than the vector control group (Fig. 4a, b). Upon SARS-CoV-2 challenge, both groups with pSARS2-S immunization also had lower viral loads and higher body weight (Fig. 4c, d). Similarly, we also explored the effect of stimulation time (3 and 5 s) on vaccine efficacy in hamsters. Spike-specific ELISA and neutralization analysis showed that 3- and 5-s stimulations delivered by the electroacupuncture machine induced neutralizing antibodies (Fig. 4e, f). Challenge studies revealed that 3 s of stimulation was sufficient to confer protection against SARS-CoV-2 infection in the hamster model (Fig. 4g, h). Based on the above results, pSARS2-S DNA is sufficient to protect hamsters from SARS-CoV-2 infection under at least 50 Hz/3-s stimulation condition delivered by the electroacupuncture machine.

**Fig. 5:**
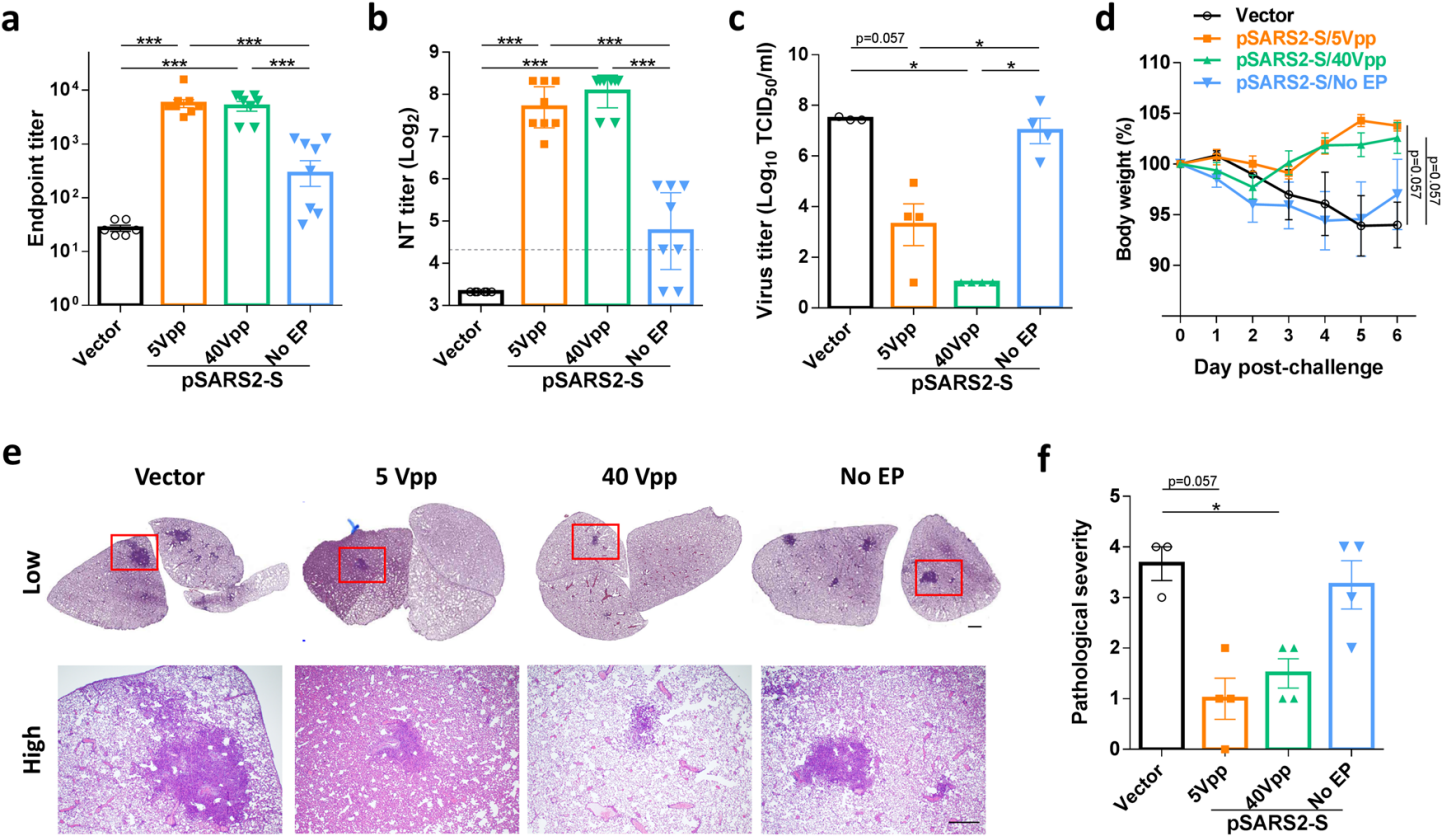
Prophylactic efficacy of the pSARS2-S DNA vaccine with or without electrostimulation against SARS-CoV-2 challenge. Syrian hamsters (*n* = 6 for vector; *n* = 8 for 5, 40 Vpp and No EP) were intramuscularly immunized twice at a 3-week interval with 100 μg of vector control, 100 μg of pSARS2-S followed by electrostimulation at the indicated voltage and 120 Hz for 5 s, or 100 μg of pSARS2-S without electrostimulation (No EP). Serum samples were collected at week 6 after the first immunization. At 3 weeks after the final immunization, Syrian hamsters were intranasally challenged with 10^5^ TCID_50_ SARS-CoV-2. **a** Spike-specific IgG antibodies were detected by ELISA. **b** Neutralization titers against wild-type SARS-CoV-2 were examined by neutralization assay. The dashed line indicates the initial fold dilution of serum samples. **c** Viral titers in the lungs of SARS-CoV-2-infected hamsters at 3 days postchallenge were determined by TCID_50_ assay. **d** The body weight change (%) of the hamsters was recorded every day after SARS-CoV-2 challenge. **e** Histopathology of lungs from infected hamsters at 6 days postchallenge was examined by hematoxylin and eosin staining. Scale bar for the low-magnification image: 1 mm, scale bar for the high-magnification image: 500 μm. **f** Pathological severity scores of infected hamsters. Neutralization titers are expressed as the geometric mean with a 95% confidence interval, and other data are presented as the mean ± SEM. *p < 0.05, ***p < 0.001 by the Mann–Whitney test.

In addition to frequency and stimulation times, we also analyzed the prophylactic efficacy in hamsters following pSARS2-S vaccination with a stimulating voltage of 5 or 40 Vpp (pSARS2-S/5 Vpp or pSARS2-S/40 Vpp) or without electrostimulation (pSARS2-S/No EP). Both pSARS2-S/5 Vpp and pSARS2-S/40 Vpp had significantly higher spike IgG antibody and neutralizing antibody titers than the vector control and pSARS2-S/No EP groups (Fig. 5a, b), and pSARS2-S/5 Vpp induced titer levels comparable to those induced by pSARS2-S/40 Vpp. Furthermore, pSARS2-S/40 Vpp vaccination resulted in a significantly lower infectious viral titer in hamster lungs, compared with the vector control and pSARS2-S/No EP groups (Fig. 5c). Both the 5 and 40 Vpp groups showed less body weight loss, with marginal significance (*p* = 0.057), than the vector group (Fig. 5d). Consistent with a previous study^16^, we found that SARS-CoV-2 infection led to several foci of inflammatory cell infiltration in hamster lungs of the vector group at day 6 postinfection (Fig. 5e). The histopathological changes in the 5 and 40 Vpp groups were smaller than those in the vector control group (lower part in Fig. 5e). Compared with that in the vector group, pathological examination revealed that the histopathological change in the 5 Vpp group was mild with marginal significance (*p* = 0.057), and the severity score in the 40 Vpp group was significantly low (Fig. 5f). Both the 5 and 40 Vpp groups had low pathological severity scores in the lungs with marginal significance (*p* = 0.057 and 0.086, respectively) relative to the pSARS2-S/No EP group (Fig. 5f). Taken together, the results highlighted the importance of electrostimulation in DNA vaccine efficacy and showed that pSARS2-S DNA delivered by an electroacupuncture machine (5–40 Vpp electrostimulation for 5 s) could be sufficient to induce the immunogenicity and efficacy of a DNA vaccine against SARS-CoV-2.

### Humoral and cellular responses induced by pSARS2-S DNA vaccine in SD rats

**Fig. 6:**
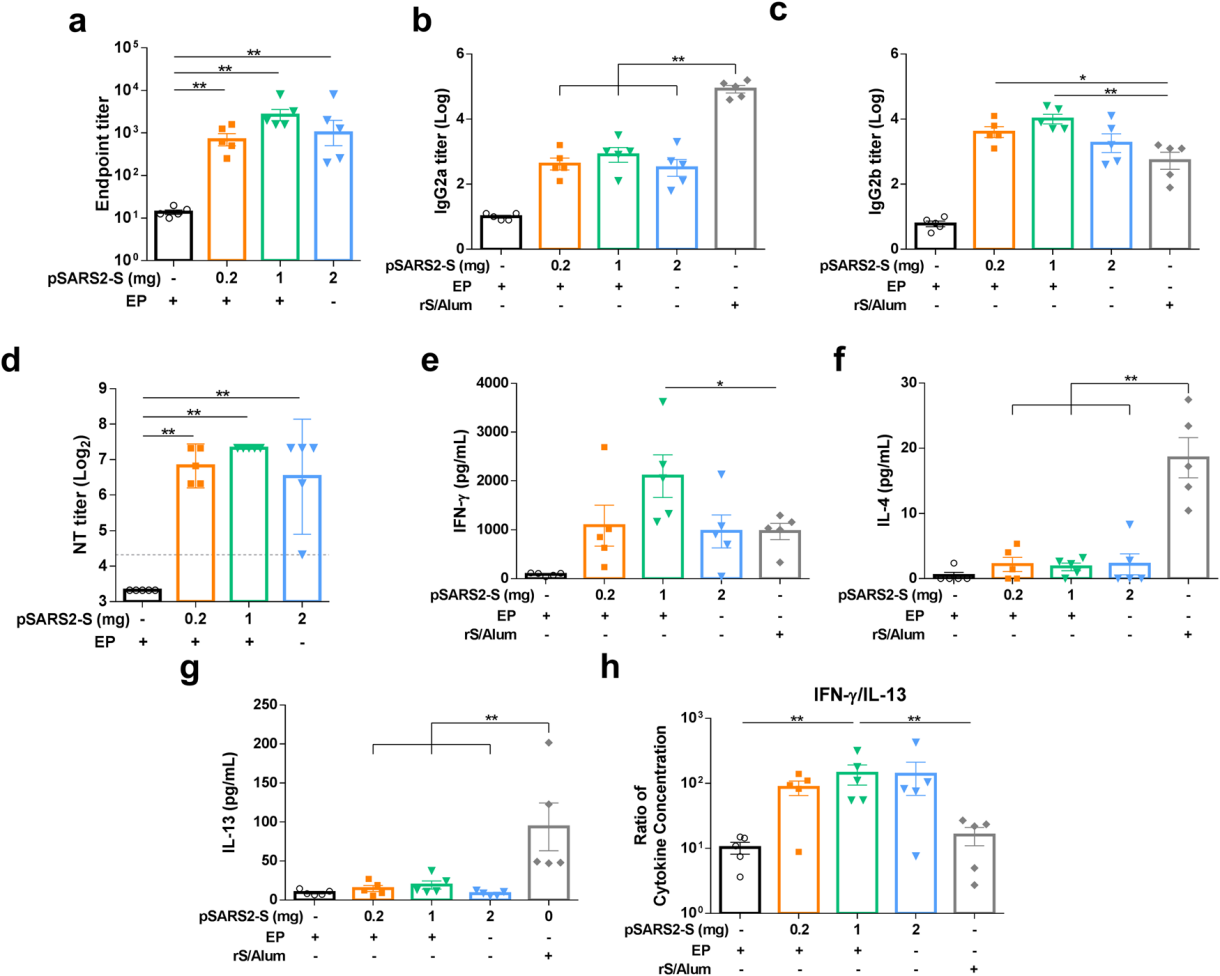
Humoral and cellular responses to the pSARS2-S DNA vaccine in a repeated-dose toxicity study. Female SD rats (*n* = 5 per group) were intramuscularly immunized three times at a 2-week interval with placebo, 0.2 and 1 mg of pSARS2-S DNA with electrostimulation (40 Vpp and 120 Hz for 5 s), 2 mg of pSARS2-S DNA without electrostimulation, or 20 μg of recombinant spike protein adjuvanted with 250 μg aluminum hydroxide (rS/Alum). Serum samples were collected at week 6 after the first immunization. **a** IgG antibodies against spike protein were measured by ELISA. **b**, **c** Immunoglobulin G subclasses (IgG2a and IgG2b) in rat serum were detected by ELISA. **d** Neutralizing activity against wild-type SARS-CoV-2 was evaluated by neutralization assay. The dashed line indicates the initial fold dilution of serum samples. **e**–**g** Splenocytes were collected at week 6 after the first immunization, and the levels of secreted IFN-γ, IL-4 and IL-13 were evaluated after restimulation with recombinant spike protein. **h** The ratio of IFN-γ to IL-13 was calculated. Neutralization titers are expressed as the geometric mean with a 95% confidence interval, and other data are presented as the mean ± SEM. *p < 0.05, **p < 0.01 by the Mann–Whitney test.

Given that pSARS2-S with electrical stimulation elicited the production of protective neutralizing antibodies against SARS-CoV-2 in mice and hamsters, we next conducted a pilot toxicity study in SD rats to evaluate vaccine immunogenicity and safety using human doses before launching GLP preclinical studies. The pilot study included an additional dose for repeated-dose toxicity evaluation. SD rats were immunized thrice with 0.2 or 1 mg of pSARS2-S with electrical stimulation or 2 mg of pSARS2-S without electrical stimulation at a 2-week interval. We examined vaccine immunogenicity again to confirm whether it was consistent with the results observed in the mouse and hamster models. Sera were collected at week 6 after the first immunization for analysis of vaccine immunogenicity. The results showed that the sera of pSARS2-S-immunized animals could indeed induce spike-specific antibodies, and the titers were 2.84 ± 0.14 and 3.42 ± 0.13 (log) in the groups with 0.2 and 1 mg of pSARS2-S/EP, respectively (Fig. 6a). The elicited antibody response persisted for at least 12 weeks after the first immunization (Supplementary Fig. 1b), which was consistent with the observation in the mouse model (Supplementary Fig. 1a). Analysis of the immunoglobulin G (IgG) subclass showed that vaccination with rS/Alum induced higher IgG2a levels (Fig. 6b), whereas immunization with 0.2 and 1 mg of pSARS2-S/EP elicited higher levels of spike-specific IgG2b antibodies (Fig. 6c). This result demonstrated that pSARS2-S/EP induced Th1-mediated subclass polarization. In addition, neutralization analysis showed that immunization with 0.2 and 1 mg of pSARS2-S/EP elicited geometric neutralization titers ranging from 113 to 160, whereas the neutralization titer was less consistent in the group that received 2 mg of pSARS2-S without EP (Fig. 6d).

To investigate the Th1/Th2 cellular immune response, we harvested splenocytes from vaccinated rats 2 weeks after the final vaccination and stimulated them with recombinant S protein (5 μg/mL) for 3 days. The secretion of the Th1-type cytokine IFN-γ (2097.6 ± 437.7 pg/mL) was high after stimulation with spike protein in the 1 mg pSARS2-S/EP immunization group (Fig. 6e), but very low levels of the Th2-type cytokines IL-4 (1.8 ± 0.58 pg/mL) and IL-13 (19.3 ± 5.14 pg/mL) were observed compared to those in the rS/Alum group (Fig. 6f, g). Furthermore, IFN-γ/IL-13 ratio analysis showed that the splenocytes from the 1 mg pSARS2-S/EP group secreted more of the Th1-type cytokine IFN-γ than the Th2-type cytokine IL-13 compared with those from the control and rS/Alum groups (Fig. 6h). These results indicated that DNA immunization can induce T cell responses that are biased toward the Th1 phenotype, confirming that the anticipated immune response occurred during the toxicity study.

**Fig. 7:**
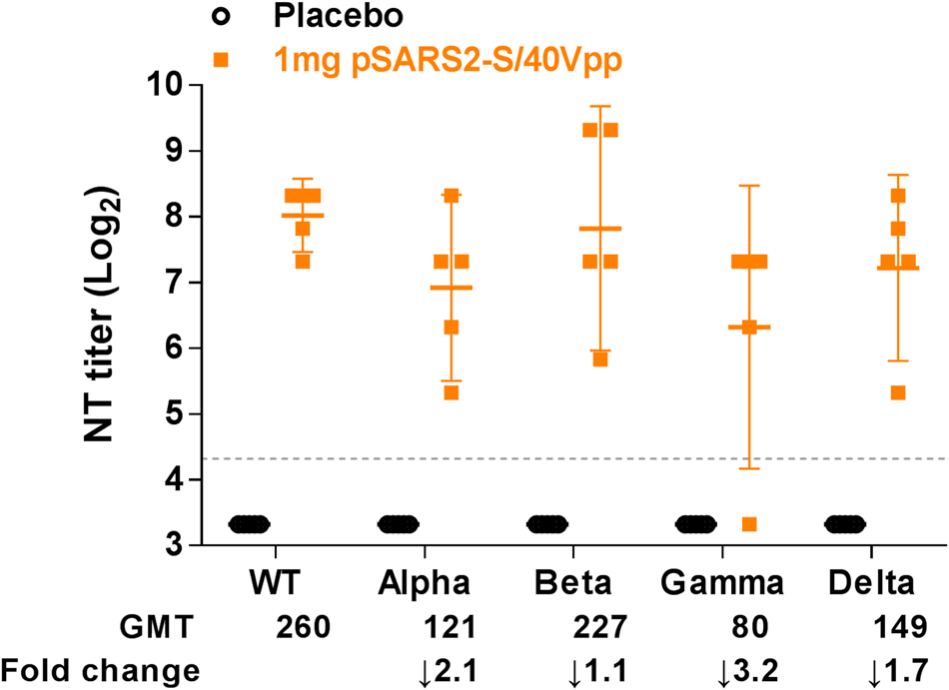
Neutralization activity of the pSARS2-S DNA vaccine against SARS-CoV-2 variants in the repeated-dose toxicity study. Male SD rats (*n* = 5 per group) were intramuscularly immunized three times at a 2-week interval with placebo and 1 mg of pSARS2-S DNA, followed by electrostimulation using an electroacupuncture machine (40 Vpp and 120 Hz for 5 s). Serum samples obtained at week 9 after the first immunization were used to evaluate the neutralizing activity against SARS-CoV-2 variants (Wuhan (WT), Alpha, Beta, Gamma, Delta) using neutralization assay. The dashed line indicates the initial fold dilution of serum samples. The neutralizing geometric mean titers (GMT) and the GMT fold change over WT strain are shown. Neutralization titers are expressed as the geometric mean with a 95% confidence interval.

Additionally, we assessed the serum neutralization activity against SARS-CoV-2 variants. The geometric neutralization titers against SARS-CoV-2 Wuhan, Alpha, Beta, Gamma, and Delta variants were 260, 121, 227, 80, and 149, respectively (Fig. 7). The fold change in neutralization titers over the Wuhan strain ranged from 1.1 to 3.2. This result indicated that 1 mg of pSARS2-S/EP exhibited a slight decrease in cross-reactivity against emergent SARS-CoV-2 variants, especially Gamma variant. Similar to Wu’s study, the neutralization activity of mouse serum induced by the Wuhan strain-based vaccine was a 2.1-fold reduction against Gamma variant^20^, and the neutralization titer was the lowest among Alpha, Beta and Gamma variants^21^. Despite variation in neutralization activity, pSARS2-S DNA vaccination is still able to induce cross-reactive neutralizing antibodies against SARS-CoV-2 variants.

### Safety assessment of DNA vaccine delivered by electroacupuncture machine

In addition to vaccine efficacy, safety concerns are a major challenge for developing DNA-based vaccines and therapies. To address these issues, hematology, serum chemistry, and DNA persistence were analyzed at 3 days after the final vaccination in the repeated-dose toxicity study in rats (*n* = 10, 5 male and 5 female). Blood biochemistry and hematology analysis revealed no significant difference between vaccine or placebo administration (Tables 2 and 3). In persistence studies, we used TaqMan real-time PCR to measure the amount of the pSARS2-S DNA vaccine in the injected muscle, skin surrounding the injection site, and draining lymph node at day 60 after the last immunization. Preliminary data showed that there were 1,046,530 copies/μg genomic DNA in the injected muscles from BALB/c mice at 24 h after vaccination and that the amount of plasmid DNA decreased to 8071 copies/μg genomic DNA at day 30 after three-dose vaccination (Supplementary Fig. 2a). At day 60 after the last immunization, the amount of remaining plasmid DNA in the injected muscle from the repeated-dose toxicity study ranged from 742 to 28,733 copies/μg genomic DNA (Supplementary Fig. 2b). Plasmid DNA in skin and lymph nodes also persisted at similar levels to that in muscle (Supplementary Fig. 2c, d). Importantly, the amount of remaining DNA in these tissues did not exceed 30,000 copies/μg genomic DNA, regardless of dose and electrical stimulation. Therefore, these data suggested that our DNA vaccine delivered by the electroacupuncture machine could have a low risk of DNA integration and meet the FDA guidance for DNA vaccines^22^.

**Table 2:** Effect of DNA vaccination on hematology in a repeated-dose toxicity study.

		Placebo		0.2 mg/EP		1 mg/EP		2 mg/no EP	
Item	Unit	Mean	SD	Mean	SD	Mean	SD	Mean	SD
WBC	10^3^/μL	10.63	3.28	13.65	3.29	12.17	3.07	12.72	3.08
NE	10^3^/μL	0.97	0.40	1.39	0.40	1.45	0.39	1.50	0.72
LY	10^3^/μL	8.84	2.79	11.35	3.02	9.73	2.96	10.02	2.16
MO	10^3^/μL	0.67	0.27	0.76	0.32	0.80	0.25	1.00	0.47
EO	10^3^/μL	0.12	0.04	0.12	0.04	0.18	0.06	0.17	0.07
BA	10^3^/μL	0.02	0.01	0.02	0.01	0.02	0.01	0.03	0.02
NE	%	9.13	2.72	10.45	3.13	12.44	3.75	11.51	3.16
LY	%	83.07	3.70	82.92	4.47	79.22	5.52	79.27	4.95
MO	%	6.38	1.59	5.58	1.75	6.68	1.73	7.70	2.46
EO	%	1.21	0.40	0.92	0.37	1.50	0.57	1.30	0.35
BA	%	0.21	0.10	0.13	0.07	0.16	0.13	0.22	0.10
RBC	10^6^/μL	7.61	1.05	7.09	0.80	7.41	0.31	7.82	0.53
HGB	g/dL	15.23	2.12	14.54	1.85	15.25	0.67	15.98	1.01
HCT	%	41.03	5.62	40.23	5.74	41.31	2.37	43.64	3.29
MCV	fL	54.00	1.88	56.55	2.40	55.79	2.06	55.87	1.94
MCH	pg	20.06	0.80	20.47	0.66	20.59	0.76	20.45	0.43
MCHC	g/dL	37.11	0.49	36.23	0.88	36.90	0.82	36.65	0.94
PLT	10^3^/μL	1095.30	134.46	1220.50	196.21	1098.10	127.26	1079.40	124.24

*WBC* White blood cell, *NE* Neutrophil, *LY* Lymphocyte, *MO* Monocyte, *EO* Eosinophil, *BA* Basophil, *RBC* Red blood cell, *HGB* Hemoglobin, *HCT* Hematocrit, *MCV* Mean corpuscular volume, *MCH* Mean corpuscular hemoglobin, *MCHC* Mean corpuscular hemoglobin concentration, *PLT* Platelet.

**Table 3:** Effect of DNA vaccination on blood biochemistry in a repeated-dose toxicity study.

		Placebo		0.2 mg/EP		1 mg/EP		2 mg/no EP	
Item	Unit	Mean	SD	Mean	SD	Mean	SD	Mean	SD
AST	U/L	86.00	23.40	110.70	12.86	89.20	17.65	88.90	21.00
ALT	U/L	32.30	4.69	34.10	3.60	32.10	3.07	35.70	4.37
γ-GT	U/L	10.00	0.00	10.00	0.00	10.00	0.00	10.00	0.00
CK	U/L	430.50	210.34	475.70	128.23	419.10	129.67	336.40	182.82
ALP	U/L	763.70	301.46	1034.10	460.26	808.20	352.47	783.40	262.26
AMY	U/L	2500.00	0.00	2452.60	105.99	2368.60	186.71	2444.80	118.46
ALB	g/dL	4.41	0.71	4.33	0.67	4.28	0.66	4.34	0.49
TP	g/dL	6.53	0.67	6.26	0.58	6.36	0.57	6.34	0.30
GLU	mg/dL	163.40	17.10	158.90	34.05	172.40	41.43	151.00	23.54
BUN	mg/dL	17.21	1.77	21.99	4.17	17.76	3.23	20.94	6.82
CRE	mg/dL	0.24	0.04	0.29	0.03	0.27	0.03	0.27	0.05
UA	mg/dL	1.67	0.15	1.74	0.22	1.57	0.36	1.62	0.61
TCHO	mg/dL	71.80	16.00	72.78	15.30	75.80	19.56	79.33	15.03
TG	mg/dL	207.20	76.83	207.10	81.74	183.70	80.14	200.40	117.63
TBIL	mg/dL	0.32	0.09	0.33	0.07	0.37	0.08	0.34	0.07
Ca	mg/dL	10.26	0.51	9.42	0.55	9.28	0.58	9.25	0.45
IP	mg/dL	5.71	0.70	5.79	1.06	6.47	1.35	6.25	1.97
Na	mEq/L	139.70	2.21	139.80	0.79	139.10	1.52	139.60	0.84
K	mEq/L	5.51	0.57	5.34	0.35	5.33	0.29	5.56	0.35
Cl	mEq/L	98.90	2.23	99.70	3.27	100.00	3.30	102.10	2.85

*AST* Aspartate aminotransferase, *ALT* Alanine aminotransferase, *γ-GT* Gamma-glutamyl transferase, *CK* Creatine kinase, *ALP* Alkaline phosphatase, *AMY* Amylase, *ALB* Albumin, *TP* Total protein, *GLU* Glucose, *BUN* Blood urea nitrogen, *CRE* Creatinine, *UA* Uric acid, *TCHO* Total cholesterol, *TG* Triglyceride, *TBIL* Total bilirubin, *Ca* Calcium, *IP* Inorganic phosphorus, *Na* Sodium, *K* Potassium, *Cl* Chloride.

## Discussion

Different types of COVID-19 vaccines have been approved or used in humans under emergency authorization^23^. Although many DNA vaccines for COVID-19 have been investigated in clinical trials, limited DNA vaccines have been approved for emergency use. DNA vaccines are thermally stable and easy to manufacture; however, further study is needed to assure the safety of new delivery devices. We previously reported the delivery of a DNA vaccine by an electroporator intended for animal use, which induced protective immunity against viral challenge in hamsters^14^. Here, we further demonstrated that a clinically used electroacupuncture machine can be used for DNA vaccination. Preclinical studies in different animals (mice, hamsters, and rats) are presented in this study. We optimized the electric pulse condition in mice and analyzed the neutralizing antibody titers (Fig. 1a–d). Only minimal damage was found in the local site after electrostimulation (Fig. 1e, f). The T cell responses were biased toward Th1 immune responses (Fig. 2). Moreover, CD8-specific T cell responses were observed in the DNA immunization group but not in the recombinant protein immunization group (Fig. 2j, k). The T cell response epitopes were identified by using overlapping synthetic peptides covering the entire sequence of the SARS-CoV-2 spike protein (Supplementary Data 1). We found that the highly reactive peptides were located in the S1 region (Fig. 3). Neutralizing antibody titers were induced in hamsters and rats (Figs. 4–6). Protective immunity was observed in hamsters infected with SARS-CoV-2 (Figs. 4, 5). Importantly, DNA vaccination can induce neutralizing antibodies against different variants, including the current variant, Delta (Fig. 7). Due to safety concerns, DNA persistence was measured in rats, and very low levels of plasmid DNA were detected in the tissues (Supplementary Fig. 2). Our results demonstrated that DNA delivery using an electroacupuncture machine is feasible for DNA vaccination.

Addressing the global threat of COVID-19 is a major challenge in public health. Although some COVID-19 vaccines have been authorized for emergency use in humans, the shortage of COVID-19 vaccines is still a problem. In particular, most vaccines need low temperatures for storage, which is a barrier for middle-income or low-income countries. DNA vaccines are stable vaccines that can be stored at room temperature for one year^24^. To facilitate DNA delivery, we used an electroacupuncture machine for DNA vaccine delivery. Electroacupuncture has been studied and used in China since the 1950s^25^, and many studies and clinical trials have been conducted around the world to evaluate the efficacy and mechanism of electroacupuncture^26–28^. Electroacupuncture machines provide low voltages (<40 Vpp) for clinical use combined with needles. We tested this idea for DNA vaccination in three different animal species, namely, mouse, hamster and rat. We found that application of 5–40 Vpp in a short time after intramuscular injection of the DNA vaccine could induce neutralizing antibody titers in three species of animals (Figs. 1, 4, 5, and 6). The DNA vaccination groups with electrostimulation had obviously higher IgG antibody titers than those without electrostimulation in mice (Fig. 1a). Similarly, DNA intramuscular injection alone induced poor IgG antibody and neutralizing antibody titers in hamsters (Fig. 5a, b), and the hamsters had high infectious viral titers and severe lung histopathology following SARS-CoV-2 infection (Fig. 5c, f) compared to the DNA vaccination groups with electrostimulation. Furthermore, we doubled the DNA dose in the no EP group of SD rats to demonstrate the necessity of electrical stimulation in enhancing DNA vaccine efficacy. The results showed that the within-group variance in the no EP group was large, especially the neutralization titer in hamster and SD rats, and some of these animals had no neutralizing antibody titer (Figs. 5b, 6d). These data suggested that electrostimulation is crucial to improve DNA vaccine efficacy and immunogenicity. To our knowledge, there are limited reports showing that electroacupuncture can be used in DNA vaccination. In this report, we showed that a low-voltage (5–40 Vpp) electric pulse after DNA intramuscular injection could increase neutralizing antibody titers (Fig. 1d) and Th1 responses (Fig. 2). Even a 5 Vpp electric pulse after DNA vaccination can induce protective immunity against SARS-CoV-2 challenge in hamsters (Fig. 5). These results indicated that DNA vaccines can be delivered by using conventional electroacupuncture to enhance immune responses for infectious diseases.

We also identified several dominant T cell response epitopes located in the RBD region of S protein using matrix pool peptides. We searched the H2-K^d^ binding sequences of the S protein using the IEDB Analysis Resource^29^. Interestingly, the highest binding score was obtained for the sequence GYLQPRTFL (a.a. 268–276), which is located in our S21 peptide (a.a. 261–285). The top 4 H2-IE^d^ sequences were within TRFASVYAWNRKRISNCV (a.a.345–362), which is located in S27 (a.a.339–363) and S28 (a.a. 352–376) peptides. S21, S27 and S28 are the peptides with the highest reactivity after DNA vaccination. A potential H2-K^d^ epitope for T cell responses, PHGVVFLHV, was observed in our identified peptide S81^30^. These results indicated that the dominant T cell epitopes in our reports include both Th and CTL epitopes. The induction of T cell-mediated immune responses by DNA vaccination is important for protective immunity. In particular, CD8^+^ T cells have been shown to play a key role in anti-SARS-CoV-2 effects. In the absence of CD8^+^ T cells, the protective effects of nucleic acid (mRNA) vaccines are reduced significantly^31,32^. These data suggested that T cell-mediated protective effects have importance beyond neutralizing antibody titers.

Electroacupuncture machines have been used in humans for a long time, and their safety is well recognized. In contrast, for other newly designed electroporators, safety needs to be proven by complex regulatory processes. Electroacupuncture machines can be used in humans immediately for DNA immunization during emergency outbreaks of infectious diseases. In this report, we demonstrated that DNA immunization via electric transfer by electroacupuncture can induce protective immunity against SARS-CoV-2 (including different variants). Electroacupuncture delivery systems provide a platform for further improvement of the safety and utility of DNA vaccines.

## Methods

### Virus titration

SARS-CoV-2 variants (hCoV-19/Taiwan/4/2020 (EPI_ISL_411927, classified as the same lineage as the Wuhan-Hu-1 reference strain (wild-type)), hCoV-19/Taiwan/792/2020 (EPI_ISL_1381386, Alpha), hCoV-19/Taiwan/1013/2021 (EPI_ISL_5854267, Beta), hCoV-19/Taiwan/906/2021 (EPI_ISL_5854262, Gamma), and hCoV-19/Taiwan/1144/2021 (EPI_ISL_5854263, Delta)) were obtained from the Centers for Disease Control in Taiwan. The virus was amplified in Vero cells (ATCC CCL-81) grown in M199 medium supplemented with 2 μg/mL TPCK-trypsin (Sigma) at 37 °C. The viral titer was determined in terms of the 50% tissue culture infectious dose (TCID_50_) using a standard method^33^. Briefly, Vero cells were seeded (2.4 × 10^4^ cells/per well) in 96-well plates and cultured in M199 medium with 5% FBS at 37 °C for 24 h to form a monolayer. The next day, serial 10-fold dilutions were prepared, and diluted virus (100 μL/well) was added to the Vero cell monolayers with six replicates per dilution. After 4 days of incubation at 37 °C, the virus-induced cytopathic effects (CPEs) in each well were recorded, and the results are expressed as TCID_50_/mL according to the method of Reed and Muench. All experiments with SARS-CoV-2 were conducted in a biosafety level 3 (BSL-3) laboratory.

### Plasmid construction and characterization

The DNA sequences encoding full-length SARS-CoV-2 spike genes (GenBank accession number: MN908947) were optimized for human codon usage and synthesized by GenScript Biotech. The gene was subcloned into the clinically used vector pVAX1 with the Kozak sequence incorporated at the 5′ end of the genes. The plasmid was transformed into *E. coli* DH5α cells for plasmid amplification. Plasmids were extracted and purified using an endotoxin-free Qiagen column system (EndoFree Plasmid Mega Kit, cat# 12381).

### Animal immunization

BALB/c mice, Syrian hamsters and Sprague-Dawley rats were obtained from the National Laboratory Animal Breeding and Research Center (Taipei, Taiwan). Mice, hamsters or rats were used between 6 and 12 weeks of age. Anesthetized mice, hamsters or rats were vaccinated with a solution containing the indicated DNA plasmid at 2- or 3-week intervals, followed by electrostimulation with 2-needle array electrodes using an electroacupuncture machine (model D0207KL, Ching Ming Medical Device Co. Ltd., Taiwan). Electrode arrays with diameters of 0.3 mm, lengths of 2.5 mm and a gap of 5 mm were used in the mouse model. For hamster and rat models, 5-mm 2-needle array electrodes that were 0.3 mm in diameter and had a 10-mm gap were used. Intramuscular EP was performed under different electric pulse times and voltages (Supplementary Table 1), which were set according to the manufacturer's instructions. A stimulation frequency of 120 Hz was used in most experiments, with the exception of Fig. 4a–d. The electrode array was brought into contact with muscle in a parallel orientation with the muscle fibers. Blood samples of mice, hamsters or rats were collected by submandibular, retroorbital or tail vein blood sampling, respectively. All animals were housed at the Animal Center of the National Health Research Institutes (NHRI) and maintained in accordance with institutional animal care protocols. All animal experimental protocols were approved by the Institutional Animal Care and Use Committee (IACUC) of the NHRI (Protocol No: NHRI-IACUC-109077-A).

### Immunoassay

The specific antibody response against SARS-CoV-2 was determined by using ELISA. Briefly, 50 μL of 4 μg/mL recombinant SARS-CoV-2 spike protein (Sino Biological, cat# 40589-V08B1) in 0.1 M carbonate buffer (pH 9.5) was coated onto 96-well microplates by overnight incubation at 4 °C. The coated plates were washed twice with 0.05% Tween 20 in PBS and then blocked with 3% BSA in PBS at room temperature for 1 h. Diluted sera from immunized animals were added to the wells and incubated for 2 h at room temperature. HRP-conjugated goat anti-mouse IgG (1:10,000; Thermo Scientific, cat# 31430), HRP-conjugated rabbit anti-hamster IgG (1:7000; Arigo Biolaboratories, cat# ARG23730), HRP-conjugated goat anti-rat IgG (1:50,000; Bethy Laboratories, cat# A110-136P), HRP-conjugated mouse anti-rat IgG2a (1:6000; GeneTex, cat# GTX02893-01) or HRP-conjugated mouse anti-rat IgG2b (1:6000; GeneTex, cat# GTX02894-01) was used as the secondary antibody. The assay was developed by using SureBlue TMB 1-Component Peroxidase Substrate (KPL, cat# 52-00-00). The absorbance was measured using an ELISA reader at 450 nm.

### SARS-CoV-2 neutralization assay

Vero cells were seeded (2.4 × 10^4^ cells/well) in 96-well plates for 24 h to form a monolayer. Preimmunization sera and antisera against the SARS-CoV-2 S protein were pretreated at 56 °C for 30 min to destroy heat-labile nonspecific viral inhibitory substances. The sera were diluted to an initial dilution of 1/20 with M199 medium, added to a well containing 200 TCID_50_ of SARS-CoV-2 in a volume of 0.2 mL, and then incubated at 37 °C for 2 h. Subsequently, the virus-serum mixture was inoculated onto Vero cell monolayers and incubated at 37 °C. Quadruplicates were prepared for each serum dilution. The CPE characteristics in each well were recorded after 4–5 days of incubation. The neutralization titer was proportional to the highest dilution of serum that prevented infection of 50% of quadruplicate inoculations. Neutralization titers below the starting dilution of 1:20 were assigned a value of 10 for calculation purposes.

### Cytokine production assay

T cell responses were assessed using cytokine ELISA. Splenocytes from immunized mice were plated at a density of 5 × 10^6^ cells per well in 24-well plates. The cells were stimulated with 5 μg/mL recombinant SARS-CoV-2 spike protein (ACROBiosystems, cat# SPN-C52H5) at 37 °C for 3 days. The supernatant was harvested and assayed for cytokine production. The levels of mouse secreted IFN-γ, IL-5, IL-13 and IL-2 were evaluated by ELISA using the matching antibody set (Invitrogen IFN-γ cat# 88-7314; IL-5 cat# 88-7054; IL-13 cat# 88-7137 and IL-2 cat# 88-7024-88) in accordance with the manufacturer’s instructions. Similarly, rat IFN-γ (Abnova, cat# KA3363), IL-13 (Abnova, cat# KA1408) and IL-4 (Invitrogen, cat# BMS628) will be quantitated by ELISA using the matching antibody set.

### ELISpot assay

BALB/c mice were injected intramuscularly twice at a 3-week interval with plasmid DNA (100 μg/mouse). Seven days after the final vaccination, mice were sacrificed, and splenocytes were collected. Splenocytes (5 × 10^5^ cells) were mixed with 10 μg/mL recombinant S1 protein (Sino Biological, cat# 40591-V08H), S2 protein (Sino Biological, cat# 40590-V08B), RBD protein (Sino Biological, cat# 40592-V08B) or peptide pools of 96 peptides overlapping by 12 amino acids from the spike protein and added to a 96-well ELISpot plate coated with anti-IFN-γ antibody (1:200; Mouse IFN-γ ELISPOT Set; BD Biosciences cat# 551083). The plates were then incubated with 5% CO_2_ at 37 °C for 48 h. After incubation, the cells were removed, and the plates were washed with 0.05% Tween-20 in PBS. A biotinylated secondary anti-IFN-γ antibody (1:250) was added to each well. After 2 h, the plate was washed, and streptavidin-HRP (1:100) was added. Spots were developed using a 3-amine-9-ethyl carbazole (AEC, Sigma, cat# 45-AEC101-1KT) solution. The spots were then counted using an ELISpot reader (Cellular Technology Ltd).

### Intracellular cytokine staining

Spleens were harvested at 7 days after the final vaccination. Single cell suspensions were collected after passing through a 70 μm cell strainer. Splenocytes were then stimulated with two S-protein peptide pools (15-mers with 11 amino acid overlap) spanning the entire SARS-CoV-2 S protein (JPT Peptide Technologies, cat# PM-WCPV-S-1), for 6 h at 37 °C in the presence of BD GolgiPlug (BD Biosciences, cat# 555029) and BD GolgiStop (BD Biosciences, cat# 554724). The cells were first washed with PBS and stained with Zombie Yellow™ Fixable Viability Dye (1:200; Biolegend, cat# 423104). Cells were then incubated with an anti-CD16/32 antibody (1:100; Biolegend, cat# 101302, clone 93) for blockade of Fc receptors before surface staining. Next, the surface stains CD3 (1:100; Biolegend, cat# 100320, clone 145-2C11), CD4 (1:100, Biolegend, cat# 100437, clone GK1.5), CD8 (1:100, Biolegend, cat# 100752, clone 53-6.7), CD44 (Biolegend, cat# 103028, clone IM7), and CD62L (Biolegend, cat# 104426, clone MEL-14) were used, and the cells were incubated for 15 min at 4 °C. After that, the cells were fixed and permeabilized using the Cytofix/Cytoperm Kit (BD Biosciences, cat# 554714) according to the manufacturer’s instructions. The cells were washed in Perm/Wash buffer and stained with intracellular staining for 30 min at 4 °C using the following antibodies: IFN-γ (1:50; Biolegend, cat# 505830, clone XMG1.2), IL-2 (1:100; Biolegend, cat# 503808, clone JES6-5H4), TNF-α (1:100; Biolegend, cat# 506321, clone MP6-XT22), IL-5 (1:100; BD Bioscienses, cat# 554396, clone TRFK5), IL-13 (1:100; Biolegend, cat# 12-7133-82, clone eBio13A), and IL-4 (1:100; Biolegend, cat# 504123, clone 11B11). All samples were acquired using an Attune NxT Flow Cytometer (Thermo Fisher Scientific), CD4^+^ and CD8^+^ T cells were gated using a hierarchical gating strategy (Supplementary Fig. 3), and CD44^hi^/cytokine^+^ memory T population was further analyzed using FlowJo software v10.6.0.

### Quantification of plasmid in tissues

Tissues were harvested, and genomic DNA was extracted by using a Biotools EasyPrep Genomic DNA Extraction Kit (DPT-BC04). To ensure the safety of the DNA vaccine, Taq-Man real-time PCR analysis was used to determine the amount of remaining plasmid DNA in tissues. Genomic DNA (50 ng/reaction) was used as a template. Real-time PCR was performed on a LightCycler^®^ 480 instrument by using 2× LightCycler^®^ 480 Probes Master (Roche Diagnostics, cat# 04707494001) and 20× customized primer-probe mixture (Topgen Biotechnology, cat# 600360CNA) (forward primer: CGTGTTTCTGGTCCTGCTG, reverse primer: GGGTGAAGGAATTAGTATAGGCG, probe sequence: TCAGTGCGTGAATCTGACA). Each PCR experiment included a standard curve generated with 10^2^, 10^3^, 10^5^ and 10^7^ copies of plasmid. The data were analyzed by LightCycler^®^ 480 software. Quantified sample copy numbers were normalized to micrograms of genomic DNA used.

### Animal challenge

Syrian hamsters (*n* = 8 per group) were intramuscularly immunized by needle injection with the indicated plasmid DNA, followed by electrostimulation as mentioned above. At 4 weeks after the last vaccination, the hamsters were challenged intranasally with 10^5^ TCID_50_ SARS-CoV-2 (hCoV-19/Taiwan/4/2020) in a 50 μL volume under isoflurane anesthesia. Their body weights (*n* = 4 per group) were recorded every day for 6 days after the challenge. Four hamsters in each group were sacrificed at day 3 after challenge for viral load quantification. To determine the viral load in the lung, left lung tissues were homogenized in 2 mL of PBS using a gentleMACS^®^ Dissociator (Miltenyi Biotec). After centrifugation at 600×*g* for 5 min, the clarified supernatant was harvested for live virus titration (TCID_50_ assay).

### H&E staining and pathologic score

BALB/c mice were electrostimulated with 0, 5, 21 and 40 Vpp for 5 s. Muscle tissues (gastrocnemius muscle) were collected at 1, 6, 24 h and 14 days after electrostimulation. The fixed muscle tissues were then embedded and sectioned before H&E staining. Microscopy examination was conducted to determine the histopathological reaction after electrostimulation. Histological analysis was evaluated according to Toxicologic Pathology^34^ using the following severity grading scheme: 1, minimal (<10%); 2, mild (10–39%); 3, moderate (40–79%) and 4, marked (80–100%). All tissue processing and histopathological evaluation were performed by Level Biotechnology Inc. (Taipei, Taiwan).

For histopathology analysis of infected hamsters, samples of lung tissue were fixed in formalin and embedded in paraffin using routine methods, and the sections were then stained with H&E. Tissue processing was performed by the Core Pathology Facility at the National Health Research Institutes (Miaoli, Taiwan). Pathological severity scores were determined by a clinical pathologist, and based on the percentage of the inflammation area for each section of right lung tissues collected from each animal in each group by using the following scoring system: 0, no pathological change; 1, infiltration area ≤10%; 2, infiltration area 10–50%; 3, infiltration area ≥50%^16^. An additional point was added when pulmonary edema and/or alveolar hemorrhage was observed. The total score for all of the lobes in the image is shown for individual animals.

### Statistical analysis

Statistical data were generated using GraphPad Prism software. The statistical significance of differential findings between experimental groups was determined by the two-tailed Mann–Whitney test. Differences were considered statistically significant if the *p* value was ≤0.05.

### Reporting summary

Further information on research design is available in the Nature Research Reporting Summary linked to this article.

## Supplementary information

Supplemental information

Supplemental data

REPORTING SUMMARY

## Data Availability

The authors declare that the data supporting the findings of this study are available within this paper or are available from the corresponding author upon reasonable request.

## References

[1] Center for Systems Science and Engineering (CSSE) at Johns Hopkins University. COVID-19 Dashboard. https://coronavirus.jhu.edu/map.html (2022).

[2] Reuters. India gives emergency approval for world's first COVID-19 DNA vaccine. https://www.reuters.com/business/healthcare-pharmaceuticals/india-approves-zydus-cadilas-covid-19-vaccine-emergency-use-2021-08-20/ (2021).

[3] Adam L (2020). Innate molecular and cellular signature in the skin preceding long-lasting T cell responses after electroporated DNA vaccination. J. Immunol..

[4] Lin F (2011). A novel prototype device for electroporation-enhanced DNA vaccine delivery simultaneously to both skin and muscle. Vaccine.

[5] Williams M (2019). Enhanced immunogenicity and protective efficacy of a tetravalent dengue DNA vaccine using electroporation and intradermal delivery. Vaccine.

[6] Bianchi G, Campanacci L, Ronchetti M, Donati D (2016). Electrochemotherapy in the treatment of bone metastases: a Phase II Trial. World J. Surg..

[7] Tebas P (2021). Safety and immunogenicity of INO-4800 DNA vaccine against SARS-CoV-2: A preliminary report of an open-label, Phase 1 clinical trial. EClinicalMedicine.

[8] Hooper J (2020). A Phase 2a randomized, double-blind, dose-optimizing study to evaluate the immunogenicity and safety of a bivalent DNA vaccine for hemorrhagic fever with renal syndrome delivered by intramuscular electroporation. Vaccines (Basel).

[9] Zia FZ (2017). The National Cancer Institute's Conference on acupuncture for symptom management in oncology: state of the science, evidence, and research gaps. J. Natl Cancer Inst. Monogr..

[10] Haidari G (2017). Combined skin and muscle vaccination differentially impact the quality of effector T cell functions: the CUTHIVAC-001 randomized trial. Sci. Rep..

[11] Yang NN (2021). Electroacupuncture ameliorates intestinal inflammation by activating alpha7nAChR-mediated JAK2/STAT3 signaling pathway in postoperative ileus. Theranostics.

[12] Zhang Z (2021). Electroacupuncture regulates inflammatory cytokines by activating the vagus nerve to enhance antitumor immunity in mice with breast tumors. Life Sci..

[13] Sher YP, Lin SI, Chai KM, Chen IH, Liu SJ (2019). Endoplasmic reticulum-targeting sequence enhanced the cellular immunity of a tumor-associated antigen L6-based DNA vaccine. Am. J. Cancer Res..

[14] Chai KM (2021). DNA vaccination induced protective immunity against SARS CoV-2 infection in hamsterss. PLoS Negl. Trop. Dis..

[15] Corbett KS (2020). SARS-CoV-2 mRNA vaccine design enabled by prototype pathogen preparedness. Nature.

[16] Imai M (2020). Syrian hamsters as a small animal model for SARS-CoV-2 infection and countermeasure development. Proc. Natl Acad. Sci. USA.

[17] Yang SJ (2021). Characterization of virus replication, pathogenesis, and cytokine responses in Syrian hamsters inoculated with SARS-CoV-2. J. Inflamm. Res..

[18] Tostanoski LH (2020). Ad26 vaccine protects against SARS-CoV-2 severe clinical disease in hamsters. Nat. Med..

[19] Brocato RL (2021). Protective efficacy of a SARS-CoV-2 DNA vaccine in wild-type and immunosuppressed Syrian hamsters. NPJ Vaccines.

[20] Wu K (2021). Variant SARS-CoV-2 mRNA vaccines confer broad neutralization as primary or booster series in mice. Vaccine.

[21] Pishesha N (2021). A class II MHC-targeted vaccine elicits immunity against SARS-CoV-2 and its variants. Proc. Natl Acad. Sci. USA.

[22] FDA Center for Biologics Evaluation and Research (CBER). Guidance for Industry: Considerations for Plasmid DNA Vaccines for Infectious Disease Indications. https://www.fda.gov/media/73667/download (2007).

[23] Zimmer, C., Corum, J., Wee, S. L. & Kristoffersen, M. Coronavirus vaccine tracker. N. Y. Times. https://www.nytimes.com/interactive/2020/science/coronavirus-vaccine-tracker.html (2022).

[24] Tebas P (2019). Intradermal SynCon(R) Ebola GP DNA vaccine is temperature stable and safely demonstrates cellular and humoral immunogenicity advantages in healthy volunteers. J. Infect. Dis..

[25] Mayor, D. F. Electroacupuncture. In: *Medical Acupuncture—A Western Scientific Approach* 2nd edn (eds Filshie, J., White, A. & Cummings, M) (Elsevier, 2016).

[26] Mayor D (2013). An exploratory review of the electroacupuncture literature: clinical applications and endorphin mechanisms. Acupunct. Med..

[27] Ulett GA, Han S, Han JS (1998). Electroacupuncture: mechanisms and clinical application. Biol. Psychiatry.

[28] Lin JG, Chen WL (2009). Review: acupuncture analgesia in clinical trials. Am. J. Chin. Med..

[29] Reynisson B, Alvarez B, Paul S, Peters B, Nielsen M (2020). NetMHCpan-4.1 and NetMHCIIpan-4.0: improved predictions of MHC antigen presentation by concurrent motif deconvolution and integration of MS MHC eluted ligand data. Nucleic Acids Res..

[30] Smith TRF (2020). Immunogenicity of a DNA vaccine candidate for COVID-19. Nat. Commun..

[31] de Alwis R (2021). A single dose of self-transcribing and replicating RNA-based SARS-CoV-2 vaccine produces protective adaptive immunity in mice. Mol. Ther..

[32] McMahan K (2021). Correlates of protection against SARS-CoV-2 in rhesus macaques. Nature.

[33] Ramakrishnan MA (2016). Determination of 50% endpoint titer using a simple formula. World J. Virol..

[34] Shackelford C, Long G, Wolf J, Okerberg C, Herbert R (2002). Qualitative and quantitative analysis of nonneoplastic lesions in toxicology studies. Toxicol. Pathol..

