# Supplementary material for: A DNA vaccine candidate delivered by an electroacupuncture machine provides protective immunity against SARS-CoV-2 infection
Source: NPJ Vaccines. 2022 Jun 3;7:60. doi: 10.1038/s41541-022-00482-0 (PMC9166770; doi:10.1038/s41541-022-00482-0)
Supplement: Supplementary file 1 — Supplemental information [file 41541_2022_482_MOESM1_ESM.pdf]

Supplementary information

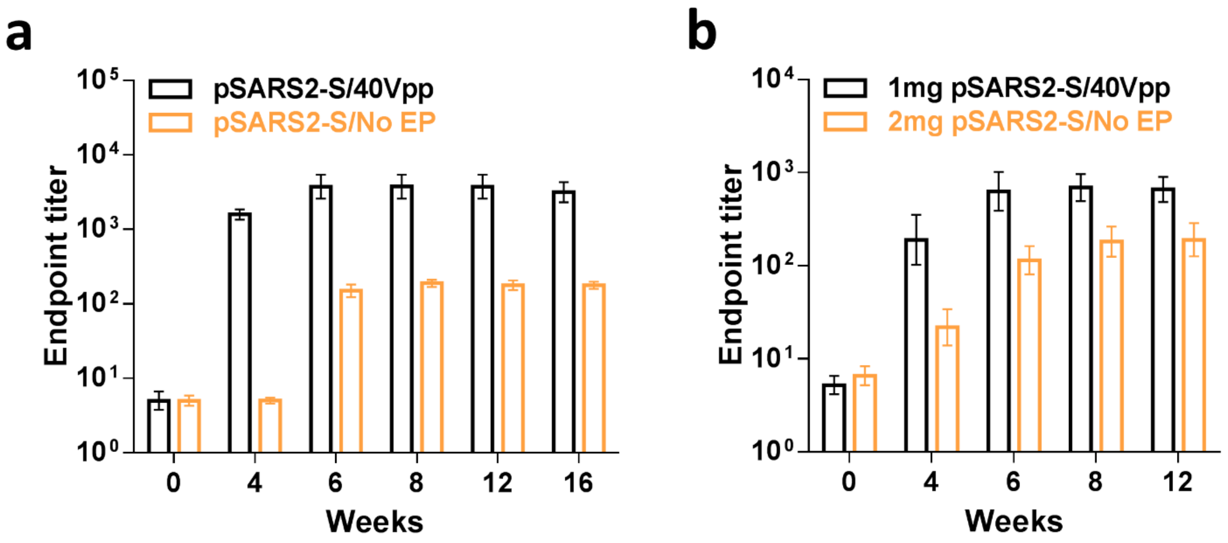

Supplementary Figure 1. Kinetics of antibody responses in mice and rats after DNA vaccination using electroacupuncture machine.

a BALB/c mice (n = 4 per group) were intramuscularly immunized twice at a 3-week interval with 100 μg of pSARS2-S DNA, followed by electrostimulation using an electroacupuncture machine (40 Vpp, 120 Hz for 5 seconds). **b** In repeated-dose toxicity study, female SD rats (n = 5 per group) were intramuscularly immunized three times at a 2-week interval with pSARS2-S DNA, followed by eletrostimulation as mentioned above. Serum samples were collected at the indicated time points after the first immunization. IgG antibodies against spike protein were assessed by ELISA. The data are presented as the mean ± SEM.

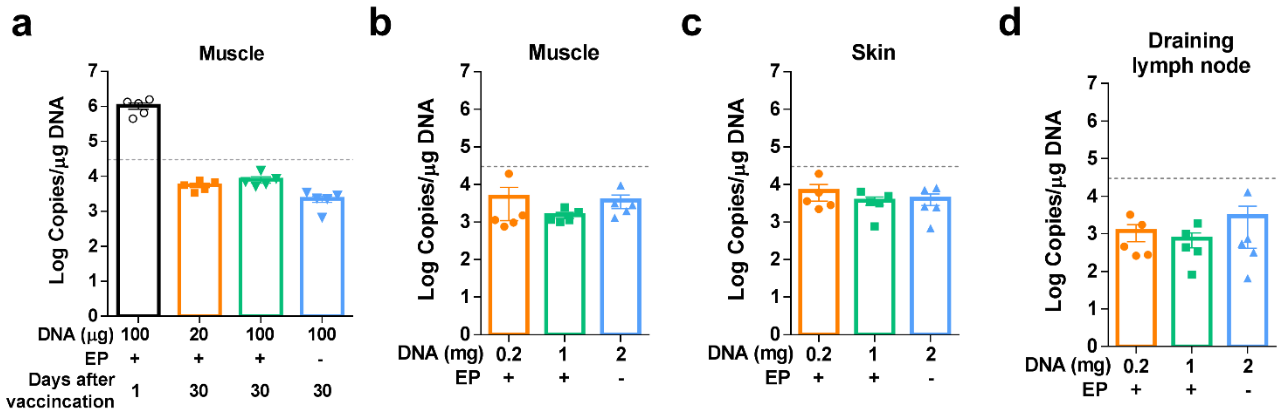

Supplementary Figure 2. Persistence of the pSARS2-S DNA vaccine in the repeated-dose toxicity study.

a BALB/c mice (n = 5 per group) were intramuscularly immunized three times at a 2-week interval with 20 or 100 μg of pSARS2-S with 40 Vpp/120 Hz electrostimulation (EP) for 5 seconds and with pSARS2-S without electrostimulation. Tissues from the site of injection (gastrocnemius muscle) were collected and processed at day 30 after the last injection. (**b**–**d**) Female SD rats (n = 5 per group) were intramuscularly immunized three times at a 2-week interval with 0.2 and 1 mg of pSARS2-S with 40 Vpp/120 Hz electrostimulation for 5 seconds and with 2 mg of pSARS2-S without electrostimulation. The gastrocnemius muscle (**b**), skin at the injection site (**c**), and draining lymph node (**d**) were collected and processed at day 60 after the last vaccination. pSARS2-S copy numbers were determined by Taq-Man real-time PCR, and the data were analyzed by LightCycler® 480 software. Quantified sample copy numbers were normalized to the amount of genomic DNA used (μg). The data are presented as the mean ± SEM.

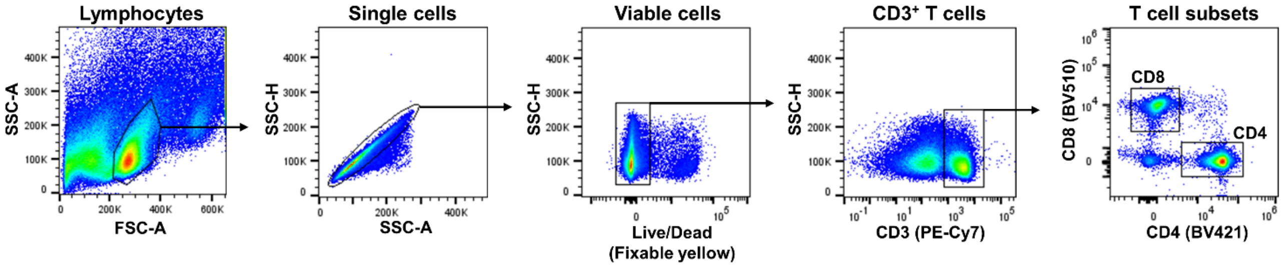

Supplementary Figure 3. Flow cytometry gating strategy to quantify CD4$^+$ and CD8$^+$ T cells.

Flowchart of the flow cytometry analysis to identify single, viable CD4$^+$ and CD8$^+$ T cells.

Supplementary Table 1. Output voltage and current.

Voltage (Vpp)	Average current (mA)
5	0.28 (120 Hz)
21	0.34 (120 Hz)
40	1.00 (50 Hz), 2.30 (120 Hz)

The pulse voltage and average current were detected at 500 ohms.
