# Supplementary material for: A DNA vaccine candidate delivered by an electroacupuncture machine provides protective immunity against SARS-CoV-2 infection
Source: NPJ Vaccines. 2022 Jun 3;7:60. doi: 10.1038/s41541-022-00482-0 (PMC9166770; doi:10.1038/s41541-022-00482-0)
Supplement: Supplementary file 3 — REPORTING SUMMARY [file 41541_2022_482_MOESM3_ESM.pdf]

nature portfolio

Corresponding author(s): Shih-Jen Liu

Last updated by author(s): Apr 5, 2022

Reporting Summary

Nature Portfolio wishes to improve the reproducibility of the work that we publish. This form provides structure for consistency and transparency in reporting. For further information on Nature Portfolio policies, see our Editorial Policies and the Editorial Policy Checklist.

Statistics

For all statistical analyses, confirm that the following items are present in the figure legend, table legend, main text, or Methods section.

n/a	Confirmed	
☐	☒	The exact sample size (n) for each experimental group/condition, given as a discrete number and unit of measurement
☐	☒	A statement on whether measurements were taken from distinct samples or whether the same sample was measured repeatedly
☐	☒	The statistical test(s) used AND whether they are one- or two-sided<br>*Only common tests should be described solely by name; describe more complex techniques in the Methods section.*
☒	☐	A description of all covariates tested
☒	☐	A description of any assumptions or corrections, such as tests of normality and adjustment for multiple comparisons
☐	☒	A full description of the statistical parameters including central tendency (e.g. means) or other basic estimates (e.g. regression coefficient) AND variation (e.g. standard deviation) or associated estimates of uncertainty (e.g. confidence intervals)
☐	☒	For null hypothesis testing, the test statistic (e.g. F, t, r) with confidence intervals, effect sizes, degrees of freedom and P value noted<br>*Give P values as exact values whenever suitable.*
☒	☐	For Bayesian analysis, information on the choice of priors and Markov chain Monte Carlo settings
☒	☐	For hierarchical and complex designs, identification of the appropriate level for tests and full reporting of outcomes
☒	☐	Estimates of effect sizes (e.g. Cohen's d, Pearson's r), indicating how they were calculated

Our web collection on statistics for biologists contains articles on many of the points above.

Software and code

Policy information about availability of computer code

Data collection	We did not use custom algorithms or software to collect data in this manuscript.
Data analysis	GraphPad Prism v.6; FlowJo software v10.6.0

For manuscripts utilizing custom algorithms or software that are central to the research but not yet described in published literature, software must be made available to editors and reviewers. We strongly encourage code deposition in a community repository (e.g. GitHub). See the Nature Portfolio guidelines for submitting code & software for further information.

Data

Policy information about availability of data

All manuscripts must include a data availability statement. This statement should provide the following information, where applicable:

- Accession codes, unique identifiers, or web links for publicly available datasets
- A description of any restrictions on data availability
- For clinical datasets or third party data, please ensure that the statement adheres to our policy

The data that support the findings of this study are available from the corresponding author, [SJL], upon reasonable request.

Field-specific reporting

Please select the one below that is the best fit for your research. If you are not sure, read the appropriate sections before making your selection.

☒ Life sciences ☐ Behavioural & social sciences ☐ Ecological, evolutionary & environmental sciences

For a reference copy of the document with all sections, see nature.com/documents/nr-reporting-summary-flat.pdf

Life sciences study design

All studies must disclose on these points even when the disclosure is negative.

Sample size	We used the prior information from our previous studies to determine the sample sizes. Basically, each group had 4-5 animals to achieve statistical power.
Data exclusions	There are no data were excluded from our analyses.
Replication	All attempts at replication were successful.
Randomization	All animals/samples are simultaneously randomized to the treatment groups without considering any other variable.
Blinding	Most of the data collection are not blinding due to the manpower limitation. The viral challenge and neutralizing assay are blinding.

Reporting for specific materials, systems and methods

We require information from authors about some types of materials, experimental systems and methods used in many studies. Here, indicate whether each material, system or method listed is relevant to your study. If you are not sure if a list item applies to your research, read the appropriate section before selecting a response.

Materials & experimental systems

n/a	Involved in the study	
☐	☒	Antibodies
☐	☒	Eukaryotic cell lines
☒	☐	Palaeontology and archaeology
☐	☒	Animals and other organisms
☒	☐	Human research participants
☒	☐	Clinical data
☒	☐	Dual use research of concern

Methods

n/a	Involved in the study	
☒	☐	ChIP-seq
☐	☒	Flow cytometry
☒	☐	MRI-based neuroimaging

Antibodies

Antibodies used

Elisa
HRP-conjugated goat anti-mouse IgG (Thermo Scientific, cat# 31430)
HRP-conjugated rabbit anti-hamster IgG (Arigo Biolaboratories, cat# ARG23730)
HRP-conjugated goat anti-rat IgG (Bethy Laboratories, cat# A110-136P)
HRP-conjugated mouse anti-rat IgG2a (GeneTex, cat# GTX02893-01)
HRP-conjugated mouse anti-rat IgG2b (GeneTex, cat# GTX02894-01)
IFN-γ mouse elisa kit (Invitrogen, cat# 88-7314)
IL-5 mouse elisa kit (Invitrogen, cat# 88-7054)
IL-13 mouse elisa kit (Invitrogen, cat# 88-7137)
IL-2 mouse elisa kit (Invitrogen, cat# 88-7024-88)
IFN-γ rat elisa kit (Abnova, cat# KA3363)
IL-13 rat elisa kit (Abnova, cat# KA1408)
IL-4 rat elisa kit (Invitrogen, cat# BMS628)

Elispot
mouse IFN-γ Elispot set (BD Biosciences, cat# 551083)

Intracellular Staining
CD16/32 (Biolegend, cat# 101302, clone 93),
CD3-PE-Cy7 (Biolegend, cat# 100320, clone 145-2C11),
CD4-BV421 (Biolegend, cat# 100437, clone GK1.5),
CD8-BV510 (Biolegend, cat# 100752, clone 53-6.7),
CD44-APC-Cy7 (Biolegend, cat# 103028, clone IM7), and
CD62L-AlexaFluor700 (Biolegend, cat# 104426, clone MEL-14)

	IFN-γ-FITC (Biolegend, cat# 505830, clone XMG1.2),<br>IL-2-PE (Biolegend, cat# 503808, clone JES6-5H4),<br>TNF-α-PerCP-Cy5.5 (Biolegend, cat# 506321, clone MP6-XT22),<br>IL-5-APC (BD Biosciences, cat# 554396, clone TRFK5),<br>IL-13-PE (Biolegend, cat# 12-7133-82, clone eBio13A), and<br>IL-4-PerCP-Cy5.5 (Biolegend, cat# 504123, clone 11B11).
Validation	The validation of the antibodies can be found in data sheet in the relevant manufactures' websites that described in the manuscript.

Eukaryotic cell lines

Policy information about cell lines

Cell line source(s)	Vero cells (ATCC CCL-81)
Authentication	Cell lines have been authenticated for the manufacturing of cGMP-grade virus vaccine in Bioproduction plant of National Health Research Institutes. We further use this cell line for SARS-CoV-2 viral culturing and neutralization assay at ABSL-3 laboratory.
Mycoplasma contamination	All cell lines were negative for mycoplasma.
Commonly misidentified lines<br>(See ICLAC register)	No commonly misidentified cell lines are in this study.

Animals and other organisms

Policy information about studies involving animals; ARRIVE guidelines recommended for reporting animal research

Laboratory animals	Female BALB/c mice or Syrian hamsters (6-12 weeks of age); male and female Sprague-Dawley rats (6-12 weeks of age)
Wild animals	There were no wild animals used in this study.
Field-collected samples	This study did not involve samples collected on the field.
Ethics oversight	All animal experimental protocols were approved by the Institutional Animal Care and Use Committee (IACUC) of the National Health Research Institutes.

Note that full information on the approval of the study protocol must also be provided in the manuscript.

Flow Cytometry

Plots

Confirm that:

☒ The axis labels state the marker and fluorochrome used (e.g. CD4-FITC).

☒ The axis scales are clearly visible. Include numbers along axes only for bottom left plot of group (a 'group' is an analysis of identical markers).

☒ All plots are contour plots with outliers or pseudocolor plots.

☒ A numerical value for number of cells or percentage (with statistics) is provided.

Methodology

Sample preparation	Spleens were harvested at seven days after the final vaccination. Single cell suspensions were collected after passing through a 70 μm cell strainer. Splenocytes were then stimulated with two S-protein peptide pools (15-mers with 11 amino acid overlap) spanning the entire SARS-CoV-2 S protein (JPT Peptide Technologies, cat# PM-WCPV-S-1), for 6 h at 37°C in the presence of BD GolgiPlug (BD Biosciences, cat# 555029) and BD GolgiStop (BD Biosciences, cat# 554724). The cells were first washed with PBS and stained with Zombie Yellow™ Fixable Viability Dye (1:200; Biolegend, cat# 423104). Cells were then incubated with an anti-CD16/32 antibody (1:100; Biolegend, cat# 101302, clone 93) for blockade of Fc receptors before surface staining. Next, the surface stains CD3 (1:100; Biolegend, cat# 100320, clone 145-2C11), CD4 (1:100, Biolegend, cat# 100437, clone GK1.5), CD8 (1:100, Biolegend, cat# 100752, clone 53-6.7), CD44 (Biolegend, cat# 103028, clone IM7), and CD62L (Biolegend, cat# 104426, clone MEL-14) were used, and the cells were incubated for 15 min at 4°C. After that, the cells were fixed and permeabilized using the Cytofix/Cytoperm Kit (BD Biosciences, cat# 554714) according to the manufacturer's instructions. The cells were washed in Perm/Wash buffer and stained with intracellular staining for 30 min at 4°C using the following antibodies: IFN-γ (1:50; Biolegend, cat# 505830, clone XMG1.2), IL-2 (1:100; Biolegend, cat# 503808, clone JES6-5H4), TNF-α (1:100; Biolegend, cat# 506321, clone MP6-XT22), IL-5 (1:100; BD Biosciences, cat# 554396, clone TRFK5), IL-13 (1:100; Biolegend, cat# 12-7133-82, clone eBio13A), and IL-4 (1:100; Biolegend, cat# 504123, clone 11B11). All samples were acquired using an Attune NxT Flow Cytometer (Thermo Fisher Scientific), CD4+ and CD8+ T cells were gated using a hierarchical gating strategy (Supplementary Fig. 3), and CD44hi/cytokine+ memory T population was further analyzed using FlowJo software v10.6.0.

Instrument	Attune NxT Flow Cytometer (Thermo Fisher Scientific)
Software	FlowJo software v10.6.0
Cell population abundance	Half a million events were acquired for rare populations analysis.
Gating strategy	Single, viable CD4+ and CD8+ T cells were gated using a hierarchical gating strategy as described in Supplementary Figure 3 in this manuscript.

☒ Tick this box to confirm that a figure exemplifying the gating strategy is provided in the Supplementary Information.
